# Recent advances in nano vehicles encapsulating cinnamic acid and its derivatives as promising anticancer agents

**DOI:** 10.1039/d5ra02640g

**Published:** 2025-06-20

**Authors:** Nadine Wafik Nabih, Mohamed S. Nafie, Asaad Babker, Hatem A. F. M. Hassan, Sherif Ashraf Fahmy

**Affiliations:** a Organic and Medicinal Chemistry Department, Faculty of Pharmacy, University of Sadat City Sadat City Menoufia 32897 Egypt; b Department of Chemistry, College of Sciences, University of Sharjah Sharjah P. O. 27272 United Arab Emirates Mohamed.ElSayed@sharjah.ac.ae mohamed_nafie@science.suez.edu.eg; c Chemistry Department, Faculty of Science, Suez Canal University Ismailia P. O. 41522 Egypt; d Department of Medical Laboratory Sciences, College of Health Sciences, Gulf Medical University Ajman United Arab Emirates; e Medway School of Pharmacy, University of Kent Chatham Maritime Kent ME4 4TB UK H.A.Hassan@kent.ac.uk; f Department of Pharmaceutics and Biopharmaceutics, University of Marburg Robert-Koch-Str. 4 35037 Marburg Germany sheriffahmy@aucegypt.edu sherif.fahmy@pharmazie.uni-marburg.de

## Abstract

Although progress in cancer diagnosis and treatment has been substantial over recent decades, several challenges remain unresolved. Among these challenges are drug resistance, the recurrence of metastatic disease, off-target toxic effects, and nonselective drug targeting. In response, increasing attention has turned to naturally derived anticancer agents that may offer both efficacy and improved safety profiles. Among these, cinnamic acid, a phenylacrylic compound abundant in Lauraceae plants such as cinnamon, has shown remarkable antitumor activity against several cancer types. However, like many phytochemicals, its clinical utility is hampered by poor water solubility, low bioavailability, and unstable pharmacokinetics. In recent years, integrating nanotechnology into drug delivery strategies has opened new avenues for overcoming these limitations. This review explores the most recent developments in the nanoformulation of cinnamic acid and its derivatives, focusing on how nanocarriers may enhance their therapeutic potential in both *in vitro* and *in vivo* cancer models.

## Abstract 2

Although progress in cancer diagnosis and treatment has been substantial over recent decades, several challenges remain unresolved.

## Graphical abstract

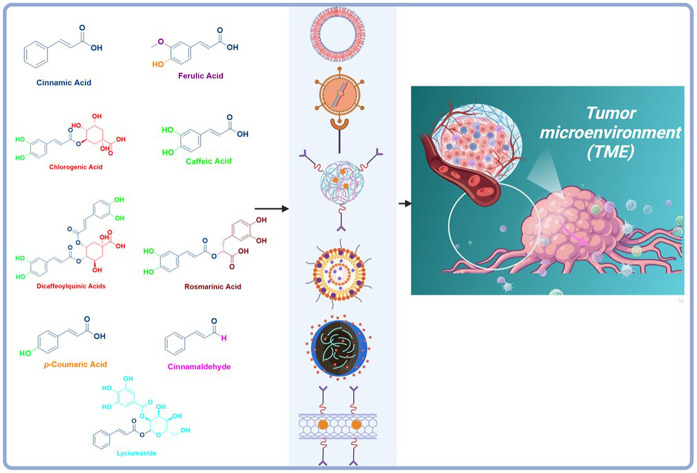

## 1 Introduction

Cancer remains one of the leading causes of death worldwide, primarily due to its aggressive proliferation, metastasis, and resistance to conventional therapies.^1–3^ Standard treatments—surgery, radiotherapy, and chemotherapy—often suffer from significant limitations, including lack of target specificity, off-target toxicities, and rapid development of drug resistance.^4,5^ As the global cancer burden continues to rise, there is an urgent need to explore alternative therapeutic strategies with improved efficacy and safety profiles.^6^

**Fig. 1:**
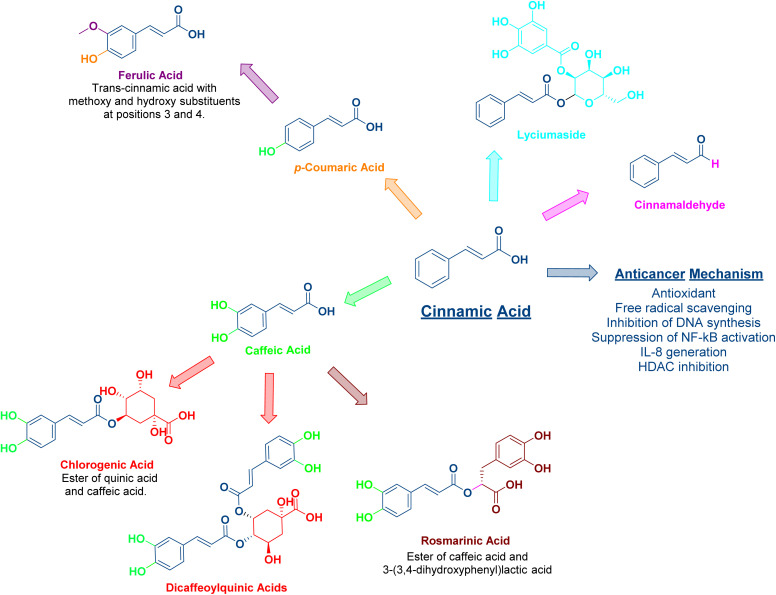
Chemical structures of the anticancer phenylacrylic acid (cinnamic acid) and its derivatives.

Phenylacrylic acids and their derivatives—naturally occurring compounds with antioxidant and pro-apoptotic properties—have recently gained attention as promising candidates in anticancer therapy (Fig. 1). These phenylpropanoids are present in various plant parts, including the fruit pericarp, leaves, and seeds.^7^ However, like many phytochemicals, their clinical utility is restricted due to poor water solubility, limited bioavailability, and metabolic instability.^8^

To overcome these challenges, nanotechnology has emerged as a transformative approach in cancer therapy. Nanoparticles (NPs) have been extensively studied for delivering natural compounds, offering several advantages such as controlled drug release, large surface area for drug loading, and enhanced solubility and absorption.^9–11^ Moreover, their biocompatibility and ability to exploit the enhanced permeability and retention (EPR) effect facilitate preferential accumulation at tumor sites, reducing systemic toxicity.^12,13^ Numerous studies have demonstrated that NP-based drug delivery systems improve phytochemicals' stability and intracellular concentration, extending their half-life and therapeutic potential.^14^

The following table discusses the key properties, including molecular weight, melting point, and aqueous solubility of cinnamic acid and its derivatives (Table 1).

**Table 1:** Physicochemical characteristics of cinnamic acid and major phenylpropanoids relevant to their pharmaceutical performance, including molecular weight (g mol^−1^), melting point (°C), and aqueous solubility (g L^−1^) at 25 °C

	Molecular weight (g mol^−1^)	Melting point (°C)	Aqueous Solubility (g L^−1^)	References
Cinnamic acid	148.16	133–135	∼0.5 at 25 °C	15
Cinnamaldehyde	132.16	−7.5	∼1.1 at 20 °C	15 and 16
*p*-Coumaric	164.16	211–212	0.39 at 25 °C	17
Ferulic acid	194.19	168–172	0.780 (pH 3.46/25 °C)	18
Caffeic acid	180.16	221–223	0.4 at 25 °C	19 and 20
Chlorogenic acid	354.31	207–209	(∼40 at 25 °C)	19 and 21
Rosmarinic acid	360.32	160–162	1.8 (pH 1.2/25 °C)	19 and 22

## 2 NPs classification

**Fig. 2:**
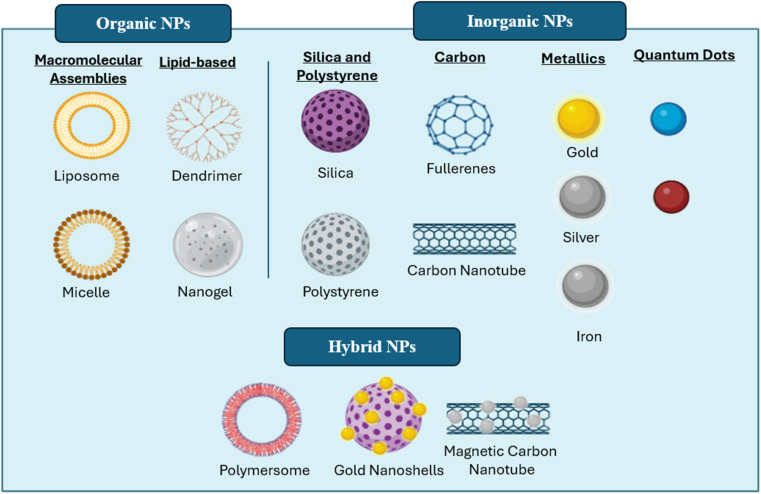
Different classes of NPs, including organic NPs (liposomes, micelles, nanogels, and dendrimers), inorganic NPs (silica, polystyrene, fullerenes, carbon nanotubes, metallics, and quantum dots), and hybrid NPs (polymersomes, gold nanoshells, and magnetic carbon nanotubes). This figure has been created by Biorender https://www.biorender.com/.

Nanoparticles used in drug delivery systems can be broadly classified into three categories: organic, inorganic, and hybrid (Fig. 2).

• Organic NPs are composed of macromolecular assemblies such as liposomes, micelles, dendrimers, and nanogels. They exhibit excellent biocompatibility and biodegradability and can be surface-functionalized with targeting ligands. However, their structural stability can be a limiting factor.^23,24^

• Inorganic NPs, including metallic nanoparticles, silica, polystyrene, and carbon-based materials (*e.g.*, fullerenes, nanotubes), possess unique optical and electronic properties and high stability. Despite these advantages, their poor biodegradability and potential toxicity pose significant challenges.

Hybrid NPs aim to harness the benefits of both organic and inorganic systems, offering enhanced targeting, stability, and biocompatibility. However, their synthesis can be complex and cost-intensive (Table 2).^25,26^

**Table 2:** Summary of the advantages and limitations of organic, inorganic, and hybrid nanocarriers used in drug delivery

Nanocarrier type	Examples	Advantages	Limitations
Organic NPs	Liposomes, micelles, dendrimers, nanogels	Biodegradable and biocompatible; surface easily functionalized; suitable for hydrophilic and hydrophobic drugs; controlled drug release	Poor physical stability; prone to leakage or fusion; limited circulation time
Inorganic NPs	Gold NPs, silica NPs, quantum dots, carbon nanotubes	High structural stability; tunable optical/electronic properties; potential for imaging and theranostics	Poor biodegradability, potential long-term toxicity, and accumulation in organs
Hybrid NPs	Lipid–polymer hybrids, MOFs, polymer-coated metal NPs	Combine the advantages of both organic and inorganic NPs; improved drug stability and targeting, and multifunctional capabilities	Complex synthesis, scalability issues, cost and regulatory concerns

## 3 Cinnamic acid

**Fig. 3:**
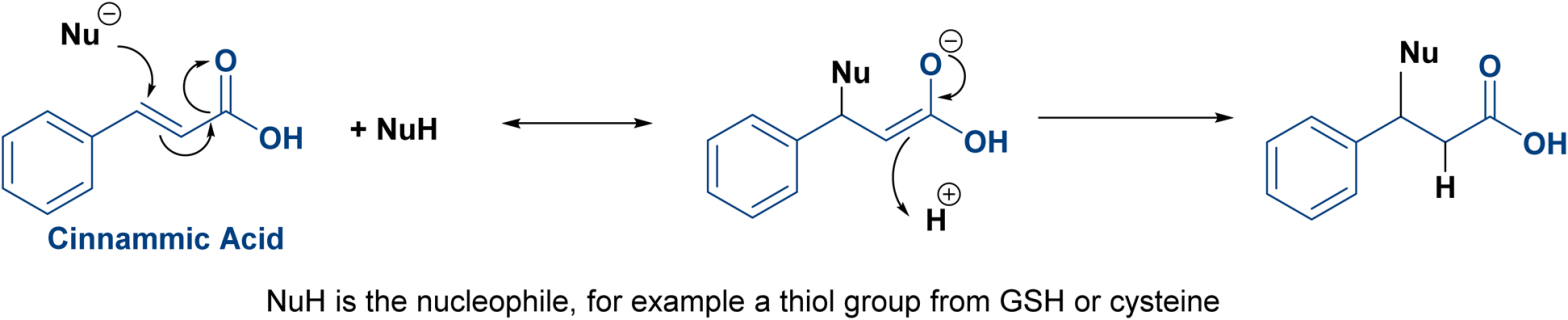
Michael addition reaction of cinnamic acid.

Cinnamic acid, or 3-phenylprop-2-enoic acid, is one of the natural constituents of the Lauraceae plants such as cinnamon. The (*E*)-isomer is much more stable than the (*Z*)-isomer; hence, 99% of the naturally occurring cinnamic acid is the (*E*)-isomer.^27^ Cinnamic acid is widely recognized for its wide range of biological properties, including antibacterial, anticancer, antioxidant, and remarkable inhibitory activity against fungi and parasites.^28^ It exerts its anticancer activity *via* several mechanisms. It inhibits DNA synthesis and NF-kB activation, generates interleukin-8, and inhibits the histone deacetylase enzyme.^29^ In addition, cinnamic acid acts as a powerful antioxidant, scavenging oxygen-free radicals; accordingly, it acts as an antiaging and immune-booster agent.^30^ Regarding cinnamic acid's structure–activity relationship (SAR), several studies have reported that the α,β-unsaturated carbonyl moiety, which is a Michael acceptor, accounts for its anticancer activity and is present in many anticancer agents since it enables interactions with cellular targets through Michael addition reactions (Fig. 3).^31^ Meanwhile, the clinical application of cinnamic acid is restricted by its inadequate stability, hydrophobicity with a very mild water solubility of 0.29 g L^−1^, ineffective membrane permeability, and poor oral bioavailability.^32^

## 4 Cinnamaldehyde

Compared to normal cells, the reactive oxygen species (ROS) levels in various cancer cells are much higher than in normal ones. As a result, cancer cells cannot withstand additional oxidative stress and express more susceptibility to ROS damage.^33,34^ Cinnamaldehyde is the principal bioactive component of the dietary spice cinnamon. It could also be obtained from cinnamic acid by controlled reduction^35^ or by its conversion into a primary alcohol, followed by mild alcohol oxidation using pyridinium chlorochromate (PDC).^36^ Besides having a SAR similar to cinnamic acid owing to the presence of a Michael acceptor pharmacophore,^37^ cinnamaldehyde exhibits excellent anticancer activity through ROS generation, ROS-mediated mitochondrial permeability transition,^33^ inhibiting tumor angiogenesis, and induction of apoptosis.^38,39^ Besides its poor bioavailability and water solubility, it is easily oxidized *in vivo*, which eventually lowers its systemic concentration.^40,41^

## 5 Hydroxycinnamic acid derivatives

**Fig. 4:**
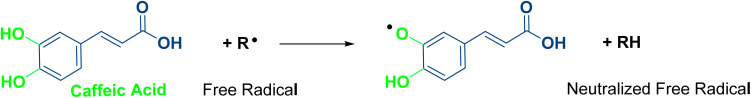
Free radical neutralization by hydroxycinnamic acid derivatives (caffeic acid).

Hydroxycinnamic acids are derived from cinnamic acid through the phenylpropanoid pathway. They are phenolic compounds that have amphipathic properties, including many compounds like *p*-coumaric acid (4-hydroxycinnamic acid), ferulic acid (4-hydroxy-3-methoxycinnamic acid), caffeic (3,4-dihydroxycinnamic acid) acid and its derivatives, and hydroxycinnamic acid esters including rosmarinic acid and chlorogenic acid. They exhibit different pharmacological properties, particularly anticancer and antioxidant activities.^42^ Regarding their relative abundance, caffeic acid is plentifully found in coffee, berries, wine, and fruits,^43^ similar to chlorogenic acid, which is found mainly in coffee, apples, pears, and blueberries,^44^ and *p*-coumaric and ferulic acid (found in fruits and vegetables).^45,46^ Meanwhile, rosmarinic acid is widely distributed in Labiatae plants, commonly found in sage, basil, peppermint, and lavender.^47^ The antioxidant characteristics of these phenolic acids are accredited to their chemical structure. The latter includes one or more free phenolic hydroxyl groups and a double bond in the aliphatic chain. Both the hydroxyl groups and the double bond enable the formation of a phenoxy radical stabilized through resonance. They can donate hydrogen atoms, which scavenge free radicals (Fig. 4). They can also modulate enzymatic activity, alter signal transduction, and activate transcription factors.^48^ Like cinnamic acid, the clinical application of hydroxycinnamic acids as free drugs is restricted due to their rapid metabolism in the peripheral circulation.^45^

## 6 Nano delivery of cinnamic acid and the other phenylpropanoids

In the following section, we will discuss the nanoencapsulation of cinnamic acid and its derivatives as pure compounds with different nanocarriers.

### 6.1 Nanoparticulate-based cinnamic acid

**Fig. 5:**
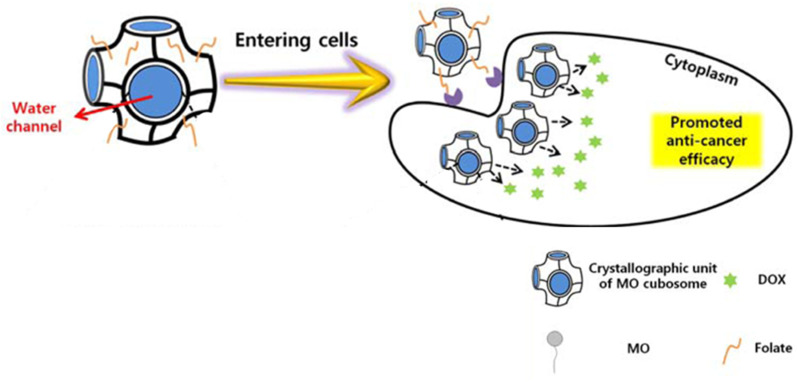
Folate-decorated MO cubosomes containing cinnamic acid and DOX with interconnected water channels. Once inside the cell, the acidic pH of cancer cells releases the drug payload directly into the cells for enhanced therapeutic efficacy. This figure has been adapted from ref. 49 permission from Springer, copyright (2020).

In 2020, Park *et al.*^49^ synthesized monoolein (MO) cubic phases by melt-hydration and micronized them into folate-decorated MO cubosomes containing poly(ethyleneimine) (PEI), cinnamic acid, and doxorubicin (DOX). MO lipids formed a bicontinuous cubic phase with interconnected water channels surrounded by lipid bilayers. These water channels served as reservoirs for hydrophilic drugs, while the bilayers encapsulated hydrophobic compounds. Cinnamic acid and PEI were self-assembled in the water channels, stabilizing the structure and enabling pH-sensitive controlled drug release. Folate molecules on the cubosome surface targeted cancer cells by binding to overexpressed folate receptors, facilitating receptor-mediated endocytosis. Once internalized, the acidic pH of cancer cells disassembled the cinnamic acid–PEI complex, releasing the drug payload directly into the cells for enhanced therapeutic efficacy (Fig. 5). According to the 3-(4,5-dimethylthiazol-2-yl)-2,5-diphenyltetrazolium bromide (MTT) assay, when tested *in vitro* on human oral epidermal carcinoma KB cells, these folate-decorated cubosomes containing DOX and PEI had higher activity compared to both folate-free cubosomes and free DOX. Yet, they showed minimal toxicity on the RAW 264.7 murine macrophage cell line. Meanwhile, flow cytometry analysis showed the strongest DOX fluorescence in KB cells treated with folate-decorated cubosomes, indicating higher cellular uptake due to folate-receptor targeting. Yet, *in vivo* studies where tumor-induced animals are treated with the designed cubosomes are still required.^49^

Recently, new PLGA NPs loaded with (*E*)-cinnamic acid (CIN-PLGA-NPs) were developed to inhibit epithelial–mesenchymal transition (EMT) in triple-negative breast cancer and control its metastasis. CIN-PLGA-NPs had a diameter of 186.3 nm, with a polydispersity index (PDI) ranging from 0.047 ± 0.072 to 0.245 ± 0.089, a zeta potential (ZP) of −28.47 mV, and an entrapment efficiency of 76.98%. The CIN-PLGA-NP optimized formulation led to enhanced cinnamic acid solubility, dissolution rate, and bioavailability. *In vitro*, they had superior cytotoxicity on MDA-MB-231 breast cancer cells with a half-maximal inhibitory concentration (IC_50_) of 0.5171 mM *versus* 2.296 mM in the cells treated with free cinnamic acid. They also increased wound healing percentage, indicating a reduced migratory potential of metastatic MDA-MB-231 cells. Furthermore, when tested in tumor animal models, the optimized NPs reduced tumor size in mice and increased necrotic and apoptotic indices, including caspase-3. As a result, CIN-PLGA-NPs exhibited superiority during *in vivo* and *in vitro* evaluation compared to free cinnamic acid, suggesting improved therapeutic outcomes and metastasis prevention in triple-negative breast cancer. Those preliminary data endow eligibility for further pre-clinical and clinical future investigations.^50^

*Staphylococcus aureus*, *Streptococcus mutans*, *Enterococcus faecalis*, and *Candida albicans* are all virulent dental pathogens implicated in the initiation and progression of oral diseases, with an impact ranging from mild conditions to chronic inflammations and the development of oral cancers, including epidermoid carcinoma. Consequently, Ravikumar *et al.*^51^ designed zinc oxide NPs capped with cinnamic acid, having an anticancer activity. Following their synthesis, zinc oxide NPs were well-dispersed and minimally agglomerated, with a particle size (PS) ranging from 100 to 200 nm. The synthesized NPs showed both, antioxidant activity confirmed by 2,2-diphenyl-1-picrylhydrazyl (DPPH) and 2,2′-azino-bis(3-ethylbenzothiazoline-6-sulfonic acid) (ABTS) assays, and antiapoptotic activity by the downregulation of Bcl-2, and upregulation of BAX and p53 apoptotic genes when tested on KB cells. Moreover, the MTT assay showed a reduction in KB cell viability by 97% following the administration of 50 μg mL^−1^ of the zinc oxide NPs.^51^

### 6.2 Nanoparticulate-based cinnamaldehyde

**Fig. 6:**
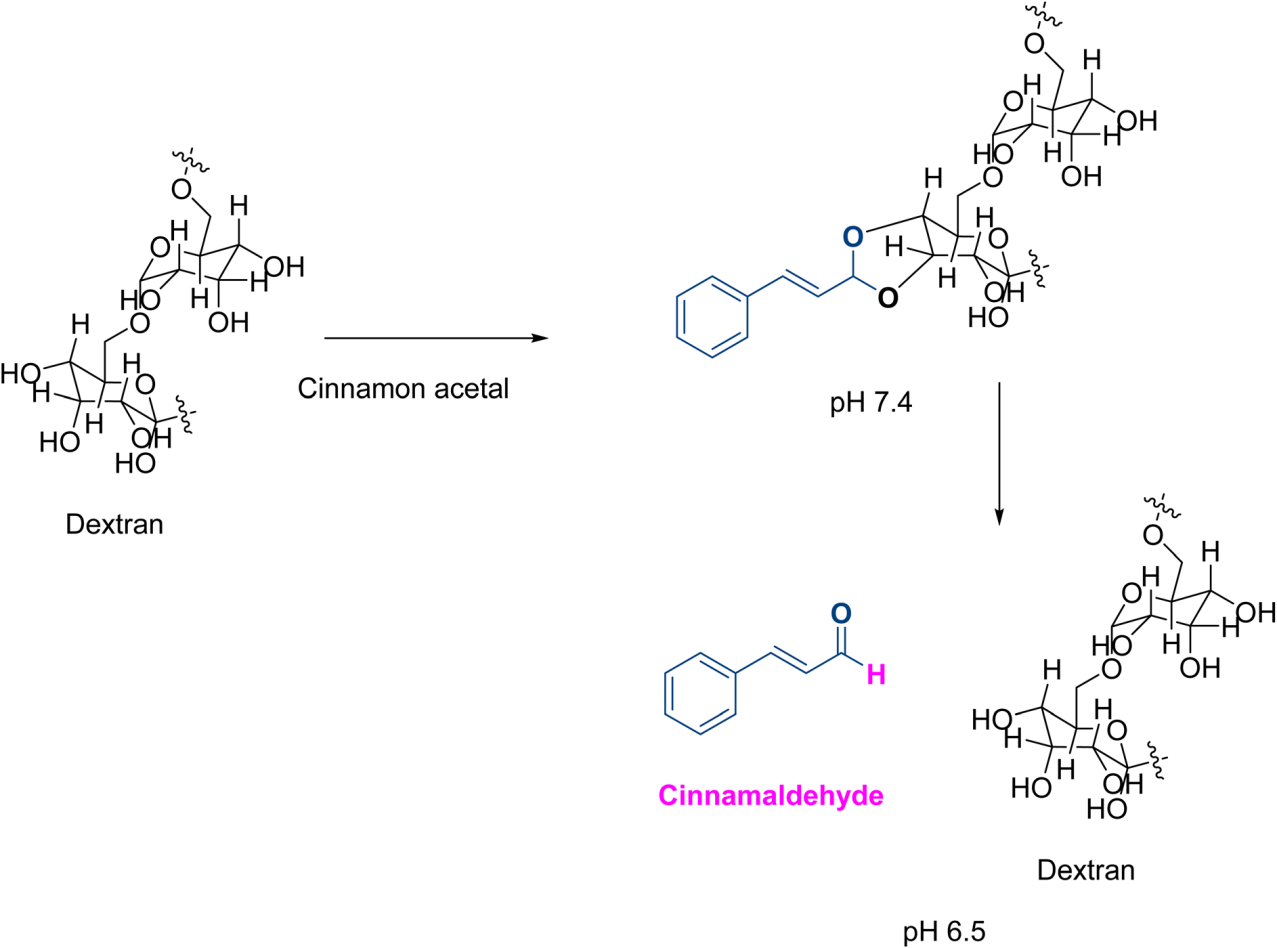
Dextran bonds to cinnamaldehyde *via* cinnamon acetal bond. The latter only breaks upon reaching a lower pH (6.5), mainly found in a cancer environment.

In 2019, Zhao *et al.*^52^ developed pH-responsive NPs encapsulating 10-hydroxy camptothecin (HCPT) by directly linking cinnamaldehyde with dextran *via* an acid-cleavable acetal bond acting against colon cancer (Fig. 6). The PS, PDI, ZP, drug loading capacity (DLC%), and encapsulation efficiency (EE%) were ∼166.4 nm, ∼0.15, ∼−16.4 mV, 21.6%, and 57.6%, respectively. Upon reaching the solid tumor environment, which is frequently characterized by a lower pH value, HCPT and cinnamaldehyde were rapidly released. They acted synergistically by generating a sharp increment in the ROS level. The MTT assay showed that the cytotoxic effect was specific to cancer cells, sparing normal cells, as demonstrated when tested on non-malignant human lung epithelial BEAS-2B cells, human vascular endothelial HUVEC cells, human cardiomyocytes 4910, and human cancer HCT-116 cells. The pH-responsive NPs notably extended the drug circulation of HCPT and cinnamaldehyde, enhanced drug accumulation in tumor sites, and exhibited excellent therapeutic performance and *in vivo* safety. Moreover, they triggered a dose-dependent increase in the expression of pro-apoptotic proteins PARP, p53, and BAX and a decrease in the expression of anti-apoptotic protein Bcl-2.^52^

**Fig. 7:**
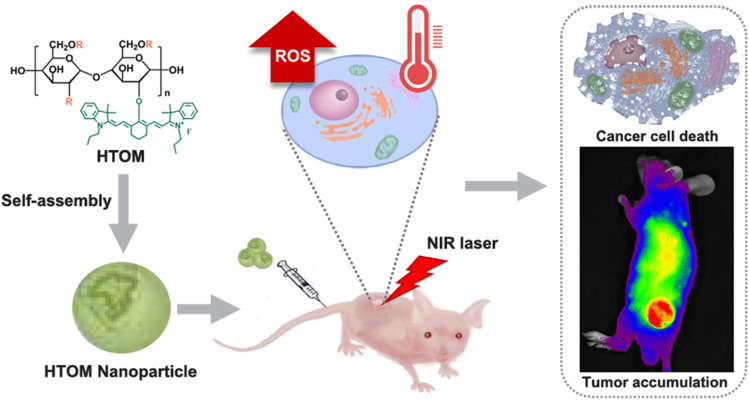
HTOM self-assembles in HTOM NPs. ROS are generated after exposure to the NIR laser, and the photo absorber IR780 converts photon energy to heat, which can selectively destroy cancer cells. This figure has been reproduced from ref. 53 permission from Elsevier, copyright (2020).

Combinational photothermal/oxidative anticancer therapy has recently gained attention in cancer therapy since tumor cells are more vulnerable to oxidative stress and heat than normal ones. Hence, Hong *et al.*^53^ developed hyperthermia and oxidative stress-inducing maltodextrin (HTOM) NPs as anticancer platforms. HTOM NPs incorporated cinnamaldehyde, as a ROS generator and oxidative stress inducer, and IR780, as a thermal stress inducer, by acid-labile acetal linkage. When combined with near-infrared (NIR) laser irradiation (808 nm), the photo absorber IR780 converts photon energy to heat capable of selectively destroying cancer cells (Fig. 7). HTOM NPs had a diameter of approximately 280 nm, a PDI of less than 1.2, and a degree of conjugation of cinnamaldehyde of ∼60%. When tested on DU145 prostate cancer cells, SW620 colorectal adenocarcinoma cells, and A549 lung adenocarcinoma cells, HTOM NPs significantly decreased tumor viability. Following their intravenous administration, they almost destroyed cancer in mouse xenograft models.^53^

**Fig. 8:**
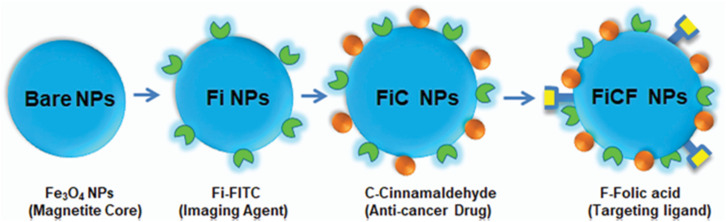
Steps of synthesis of FiCF NPs: Fe_3_O_4_ NPs were functionalized with imaging agent FITC, loaded with cinnamaldehyde, and decorated with folic acid as the targeting ligand. This figure has been reproduced from ref. 41 permission from NJC, copyright (2021) https://creativecommons.org/licenses/by/3.0/.

Taking advantage of its anticancer potential, Shetty *et al.*^41^ loaded cinnamaldehyde onto magnetic (Fe_3_O_4_) NPs that were functionalized with fluorescein isothiocyanate (FITC) and folic acid (FiCF NPs) (Fig. 8). Magnetic NPs were utilized for imaging and active drug targeting in breast cancer cells. Their PS was approximately 10 nm, with a ZP of −59.6 mV, they were evenly dispersed, and 20% of cinnamaldehyde was loaded on the surface. As mentioned earlier, folic acid is responsible for enhanced NP internalization into cancer cells and their accumulation in the cytoplasm and nucleus. When tested on breast cancer lines MCF-7 and MDAMB-231, at 20 μg mL^−1^, FiCF NPs decreased their viability up to 15.88 0.85% and 49.56 0.65% with an IC_50_ of 2.84 and 17.44 μg mL^−1^. Interestingly, no toxic effect was detected on the non-cancerous breast MCF-10A cell line. Moreover, FiCF NPs promoted cancer apoptosis *via* increased expression of generic caspases (caspase-1, -3, -4, -5, -6, -7, -8, and -9) in a dose-dependent manner. They also decreased the tumor burden in the mouse breast cancer model, prolonged circulation time, enhanced hydrophilicity, and sustained release of cinnamaldehyde. Additional safety studies are still necessary before advancing their potential for clinical application.^41^

Lu *et al.*^54^ employed a prodrug strategy and synthesized pH-responsive NPs loaded with cinnamaldehyde and phenylboronic pinacol ester-caged CHPT prodrug (ProCPT). The NPs were made from amphiphilic block copolymers *via* reversible addition–fragmentation chain-transfer (RAFT) polymerization. It consisted of PEG, cinnamaldehyde-conjugated methacrylate (CAMA), responsible for the release of cinnamaldehyde in acidic environments, and imidazolyl and alkyl moiety-containing methacrylate (ImOAMA) that helped with endosomal escape. These components were self-assembled into micelles in water, forming stable NPs for drug delivery. The pH-responsive NPs had a PS, a PDI, a drug loading efficiency, and a DLC% of 54.3 nm, 0.25, 72.8%, and 10.3%, respectively. Under an acidic environment, cinnamaldehyde is released from NPs and increases ROS levels, activating the anticancer prodrug. Both *in vitro* and *in vivo* evaluations showed that the pH-responsive NPs could efficiently induce tumor apoptosis and inhibit cancer growth with minimal adverse effects on normal tissues. CCK-8 assay revealed a strong cytotoxic effect. The IC_50_ values were determined to be the CPT-equivalent concentrations 4.6, 3.9, and 3.3 μM, respectively, against the three tested cell lines: 4T1 metastatic breast cancer cells, A549, and HeLa cervical cancer cells. Moreover, the comet assay detected potent DNA destruction capability mediated by the ROS generation.^54^

**Fig. 9:**
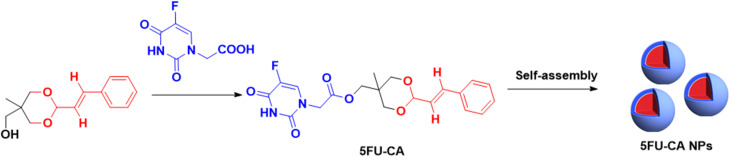
Cinnamaldehyde acetal reacts with 5FU–COOH to obtain 5FU–CA that self-assembles into 5FU–CA NPs.

Intending to attenuate off-target toxicity and achieve a high therapeutic effect, Fang *et al.*^55^ developed carrier-free self-assembled nano-drug particles of 5 fluorouracil (5FU) and cinnamaldehyde, designated 5FU-CA NPs, attached *via* acetal and ester bonds, with a PS of 170 nm, a PDI of 0.121, and a uniform size distribution (Fig. 9). *In vitro* testing revealed that 5FU–CA NPs were efficiently internalized by HepG2 liver cancer cells. The slow antimetabolic activity exerted by 5FU was complemented by the fast-acting effect of cinnamaldehyde, which mediated cancer cell destruction by raising the level of ROS and cytochrome C (cytC). Additionally, MTT cytotoxicity testing on HpeG2 cells revealed that 5FU–CA NPs exerted a synergistic anticancer activity following the cleavage of the acetal and ester bonds, leading to increased cell-killing efficiency and apoptosis, and a decrease in the IC_50_ value compared to free 5FU. Meanwhile, when tested on the H22 murine hepatoma cell line, 5FU–CA NPs had an IC_50_ of 21.291 μM at 48 hours compared to 66.903 μM for free 5FU and 142.755 μM for free cinnamaldehyde. No significant toxicity was observed in the QSG human hepatocellular cell line. Moreover, *in vivo* studies demonstrated superior tumor growth inhibition and reduced systemic toxicity following administration of 5FU–CA nanoparticles, surpassing the effects achieved with the combined administration of free 5FU and cinnamaldehyde.^55^

Qi *et al.*^56^ developed a pH-sensitive amphiphilic block copolymer NP for controlled drug delivery with enhanced anticancer effects. These NPs were synthesized using the atom transfer radical polymerization (ATRP) technique and consisted of a hollow mesoporous silica (HMS) core modified with C18 alkyl chains, coated with a pH-sensitive polymer, and loaded with DOX. Cinnamaldehyde moiety was incorporated into the polymer through the pH-sensitive monomer 2-styryl-1,3-dioxan-5-yl methacrylate (SDMA) containing a cinnamic aldehyde acetal group. The designed NPs had a narrow PDI ranging from 1.33 to 1.44, and a DLC% of 36.7%. *In vitro* studies showed selective inhibition of human melanoma cells A375 over normal skin fibroblast GM. This was attributed to the release of cinnamaldehyde in the acidic tumor environment following pH-dependent acetal group hydrolysis, enhancing the anticancer effect alongside DOX. In addition, the NPs also enabled fluorescence imaging, aiding in tracking cellular uptake and drug release.^56^

**Fig. 10:**
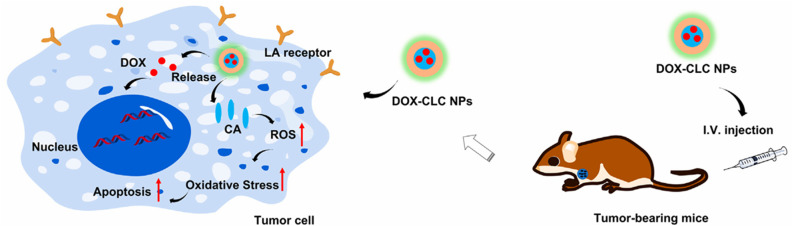
Following their administration in tumor-bearing mouse models, CLC-DOX NPs exhibited both active tumor-targeting capability and penetration (due to the presence of LA), and increased ROS levels by cinnamaldehyde, which induced apoptosis, acting synergistically with the anti-tumor drug DOX. This figure has been reproduced from ref. 57 permission from Frontiers, copyright (2022). The figure has been cropped for clarity. Deed – Attribution 4.0 International – Creative Commons (https://creativecommons.org/licenses/by/4.0/deed.en).

A hybrid NP based on lactobionic acid (LA) modified chitosan and cinnamaldehyde-modified chitosan was prepared by Zhou *et al.*^57^ as a delivery system for DOX. These CLC-DOX NPs (cinnamaldehyde-LA-chitosan hybrid NPs loaded with DOX) exhibited both active tumor-targeting capability and penetration (due to the presence of LA), and ROS regulation by cinnamaldehyde, acting synergistically with the anti-tumor drug DOX and improving its stability and half-life in blood circulation (Fig. 10). The hybrid NPs had a diameter of 338 nm and a PDI of 0.164. The *in vitro* cytotoxicity tests on HepG2 and H22 cell lines using the MTT assay, along with the *in vivo* antitumor study, revealed that the designed nanoparticles (NPs) exhibited superior anticancer activity compared to free doxorubicin (DOX). Specifically, only 21% of HepG2 cells and 51.1% of H22 cells survived after treatment with the NPs, demonstrating their enhanced efficacy in killing cancer cells. This is attributed to the oxidative stress exerted by the released cinnamaldehyde, which enhances cancer cell apoptosis and acts side by side with the direct cytotoxic anticancer effect of DOX, following NPs intracellular depolymerization.^57^

As mentioned earlier, owing to their mutually reinforced property, combining chemodynamic therapy (CDT) with photodynamic therapy (PDT) has gained significant attention. Consequently, Bai *et al.*^58^ loaded ferrocene–cinnamaldehyde conjugates (Fc–CA) into porphyrin-based MOF (specifically PCN-224) NPs coated with hyaluronic acid (HA), producing supramolecular NPs (Fc–CA-PCN-HA) to raise the level of hydrogen peroxide (H_2_O_2_) in tumor cells and to amplify the therapeutic effects. The HA coating enables CD44 receptor-mediated internalization, allowing cancer cells to selectively uptake (HeLa and A549). Once inside tumor cells, the acidic pH micro-environment dissociates the NPs, releasing cinnamaldehyde and ferrocene. In turn, cinnamaldehyde activates NADPH oxidase, leading to increased H_2_O_2_ production inside cancer cells, and ferrocene catalyzes the conversion of H_2_O_2_ into hydroxyl radicals (˙OH) *via* the Fenton reaction, enhancing CDT. Simultaneously, Fenton's reaction produces oxygen, relieving tumor hypoxia and enhancing PDT by generating singlet oxygen (^1^O_2_). Given the cascade reactions induced therapeutic performance of Fc–CA-PCN-HA *in vitro* and *in vivo*, the H_2_O_2_-elevation strategy improves ROS-mediated cancer cell apoptosis, making PDT/CDT more effective. The calculated PS and ZP were 90 nm and −22.5 mV, respectively. CCK-8 cytotoxicity assay proved that Fc–CA-PCN-HA with light irradiation had the highest cytotoxicity on HeLa cells compared to PCN-HA alone, PCN-HA with light irradiation, and Fc–CA.^58^

### 6.3 Nanoparticulate-based *p*-coumaric acid and ferulic acid

In 2020, SLNs were loaded with (*E*)-resveratrol and ferulic acid, coated with chitosan, and conjugated with folic acid. Solvent evaporation and hot homogenization methods were used for the SLN formulation, while folic acid was conjugated *via* the co-encapsulation method of stearic acid. The aim was to evaluate the SLNs' colon-targeting efficacy and anticancer potential. The nano vehicles showed interesting stability in acidic pHs, a PS of 174 ± 5 nm, a PDI of 0.166, and a ZP of −25.9 mV. MTT assay conducted on HT-29 (colon adenocarcinoma) cells showed an IC_50_ of 10 μg mL^−1^, indicating potent cytotoxicity. The cancer cell targeting and apoptosis studies confirmed that the cytotoxic effect was exclusive to cancer cells without causing harm to normal NIH3T3 mouse embryonic fibroblast cells.^59^

Rajendran *et al.*^60^ used ferulic acid as a reducing and stabilizing agent to synthesize gold NPs (Fa-AuNPs) as a potential therapeutic agent for skin cancer. Fa-AuNPs had a mean PS, a PDI, and a ZP of 34.2 ± 1.3 nm, 0.137, and −28.5 mV, respectively. When tested in human skin cancer cells (A431) and normal keratinocytes (HaCaT cells), a dose and time-dependent cytotoxic effect was witnessed in A431 cells while HaCaT cells were intact. The IC_50_ value was calculated to be 168.7 ± 2.3 μg mL^−1^ for Fa-AuNPs. They effectively induced apoptosis, as evidenced by the sub-G1 population, exhibited strong ROS generation, and significantly enhanced caspase-3 activity. Cam assay, which is considered a reliable *in vivo* model to evaluate angiogenesis, substantiated that the Fa-AuNPs had a high ability to inhibit angiogenesis *in vivo*.^60^

To improve *p*-coumaric acid bioavailability, Mariadoss *et al.*^45^ designed an aptamer-guided drug-delivery system targeting triple-negative breast cancer (MDA-MB-231). The encapsulation of *p*-coumaric acid was achieved using aminated starch by forming an amide bond, aiming to enhance its biocompatibility and bioavailability. The resulting *p*-coumaric acid-loaded aptamer conjugated starch NPs (Apt-*p*-CA-AStNPs) had a PS, a ZP, a PDI, and an EE% of 218.97 ± 3.07 nm, 29.2 ± 1.35 mV, 0.299 ± 0.05, and 80.30 ± 0.53%, respectively. The presence of ligand-like aptamer enabled targeted drug delivery of *p*-coumaric acid to MDA-MB-231 cells by specifically binding to nucleolin, a receptor overexpressed on cancer cells. After their administration, Apt-*p*-CA-AStNPs showed a rapid and bursting release followed by a sustained and constant release that lasted around 42 hours, enhancing the anti-cancer activity. They didn't cause any adverse effects on the non-cancerous NIH3T3 cell line. On the other hand, the aptamer-tagged NPs induced cytotoxicity in MDA-MB-231 cells with an IC_50_ value of 22.56 ± 1.97 μg mL^−1^ by regulating ROS levels, causing nuclear damage, altering mitochondrial membrane potential, and inducing apoptosis-related protein expression.^45^

**Fig. 11:**
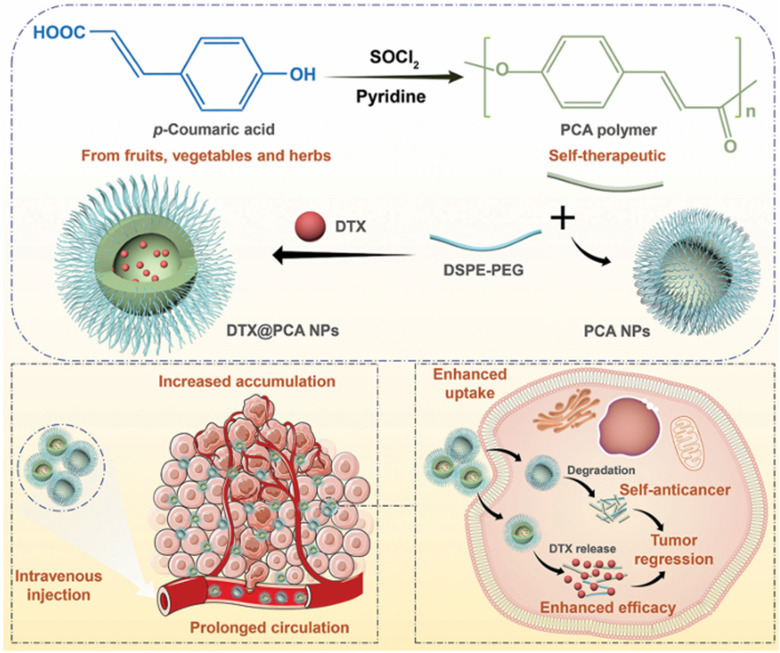
Illustration showing the synthesis of PCA from *p*-coumaric acid. PCA was then formulated into NPs loaded with DTX. DTX@PCA NPs exhibited antiproliferative activity and antimetastatic ability, enhanced cellular uptake by cancer cells, and increased tumor cell accumulation. This figure has been reproduced from ref. 61 permission from RSC, copyright (2022).

As a promising candidate for the development of self-therapeutic biomaterials, Wang *et al.*^61^ developed biocompatible polymers consisting of poly(*p*-coumaric acid) (PCA) from *p*-coumaric acid. PCA was formulated into NPs through the nanoprecipitation method. Then, the NPs were loaded with Docetaxel (DTX) to form DTX@PCA NPs. The PS, PDI, ZP, EE%, and DLC% were calculated as follows: 83.4 ± 4.1 nm, ∼0.2, −20.4 ± 0.4 mV, 33.4 ± 1.3%, and 5.57 ± 0.22%, respectively. MTT *in vitro* cytotoxicity assays performed on CT26 colon tumor cells exhibited a dose-dependent antiproliferative activity, antimetastatic ability, and enhanced cellular uptake by tumor cells. The latter findings were also confirmed by the three-dimensional tumor spheroid assay. *In vivo*, DTX@PCA NPs were characterized by preferential tumor accumulation, prolonged systemic circulation, and reduction of DTX side effects. In addition, blank PCA NPs themselves demonstrated additional cancer inhibition activity to some extent with high safety, highlighting the potential of PCA as a promising self-therapeutic nanocarrier for anticancer drug delivery (Fig. 11).^61^

**Fig. 12:**
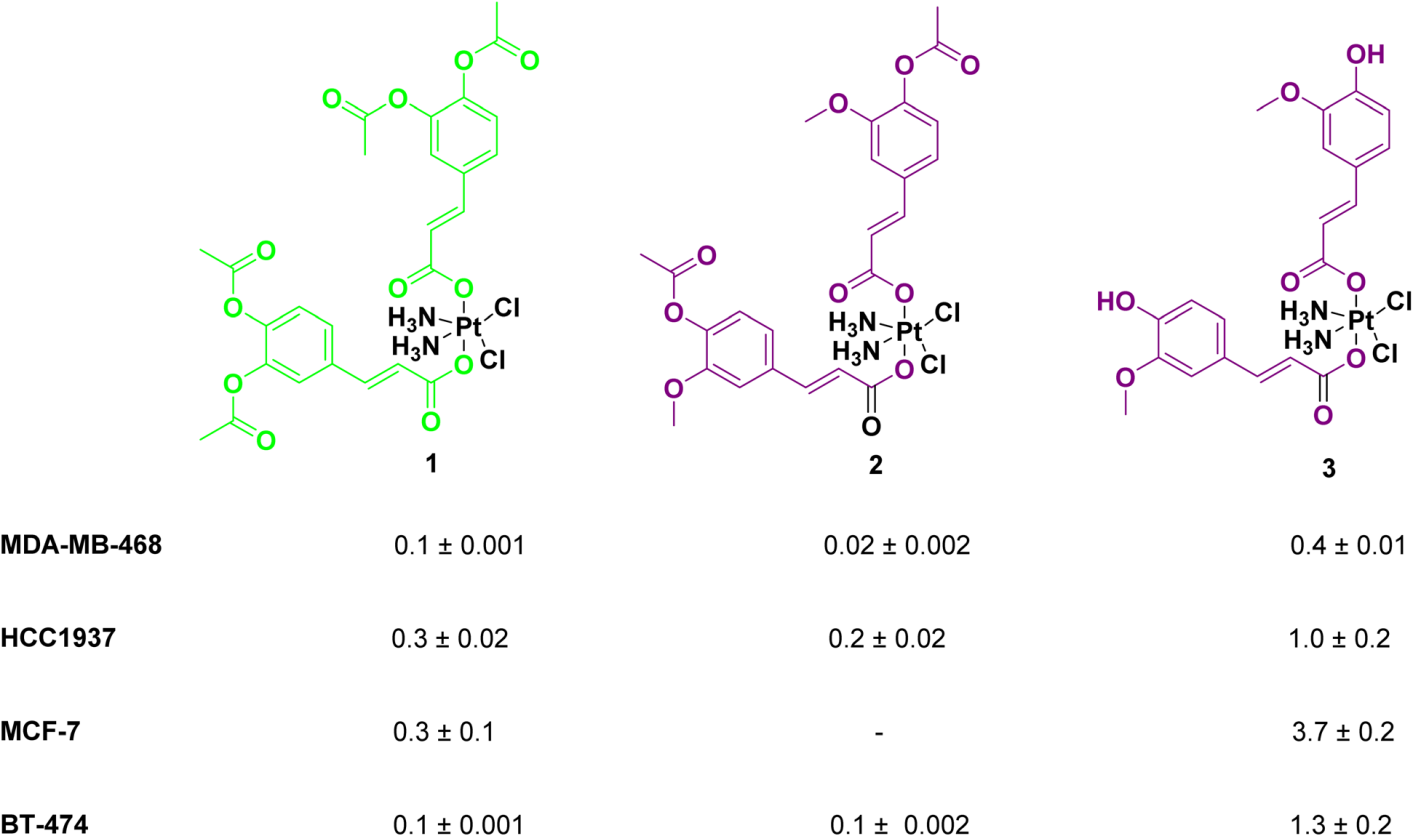
The three cisplatin derivatives bearing acetyl-protected caffeate (1), acetyl-protected ferulate (2), and ferulate (3) as axial ligands, and their corresponding IC_50_ (μm) values on the 4 breast cancer cell lines.

Creating platinum(iv) complexes *via* the introduction of active ligands in the axial positions of platinum(ii)-based antineoplastic agents (like cisplatin) to construct multifunctional prodrugs is a recent and tempting strategy. It addresses several limitations encountered during the platinum(ii) therapy that are used to limit its clinical efficacy, like drug resistance and significant side effects. In this concept, Predarska *et al.*^62^ designed three cisplatin derivatives bearing acetyl-protected caffeate, acetyl-protected ferulate, and ferulate as axial ligands (Fig. 12) loaded into SBA-15 mesoporous silica NPs (MSN) with an EE% of 70.4%, 83.8%, and 89.2%, respectively, and a DLC% of 7.51%, 9.23%, and 10.33%, respectively. A narrow size distribution of the particles was observed, ranging between 200–400 × 600–800 nm. Under simulated physiological conditions, they facilitated a controlled, moderate-to-slow release of platinum(iv) compounds, ensuring prolonged drug exposure after tumor accumulation. MTT cytotoxicity assay showed anticancer prospects for all three MSNs with higher cytotoxicity compared to cisplatin in four breast cancer cell lines (BT-474, MCF-7, MDA-MB-468, and HCC1937). Among the three derivatives, acetyl-protected caffeate was the most potent, demonstrating the ability to suppress cell growth by inhibiting proliferation and inducing apoptosis, and sparing treated mice from nephrotoxicity generally observed during cisplatin treatment.^62^

The DOX/cyclophosphamide regimen is widely recognized for its cancer resistance and numerous side effects. Hence, Helmy *et al.*^63^ proposed combining DOX with (*E*)-ferulic acid encapsulated in folate-receptor-targeted PLGA NPs and testing their anticancer efficacy and safety profile. The obtained NPs had a diameter of 148 ± 6 nm, a ZP of 6.63 ± 1.3 mV, and an entrapment efficiency of (*E*)-ferulic acid of 73 ± 2%. When tested *in vivo* on a breast cancer mouse model, the synthesized NPs inhibited Notch signaling. They also repressed Notch synergy with Wnt, estrogen, progesterone, human epidermal growth factor receptor 2 (HER2) pathways, and P-gp level. Meanwhile, heart, bone, and liver health, as well as WBC count, were preserved. Consequently, it was concluded that the NPs reduced the side-effects of DOX and cyclophosphamide while exerting anticancer activity surpassing the regimen itself.^63^

Another attempt to target colon cancer was made by El-Gogary *et al.*^64^ through encapsulating ferulic acid into lipidic nanocapsules (NCs). The latter's characterization showed a mean diameter of 36.8 nm, a PDI less than 0.2, a ZP of −12.2 mV, and an EE% of 89.8%. MTT assay conducted on colon cancer cell lines Caco-2 cells and HCT-116 confirmed the NCs' significantly high cytotoxicity. *In vivo* evaluation showed that ferulic acid lipid NCs had antioxidant and anti-inflammatory activities. The autoregulation of BAX/Bcl-2 genes also confirms lipidic NCs' strong apoptotic capacity. However, comprehensive safety evaluation of both the nutraceutical and lipid-based nanocapsule systems across multiple organs, including immunohistochemical analysis of tissue sections, remains necessary to confirm the biocompatibility of these nanocarriers fully.^64^

**Fig. 13:**
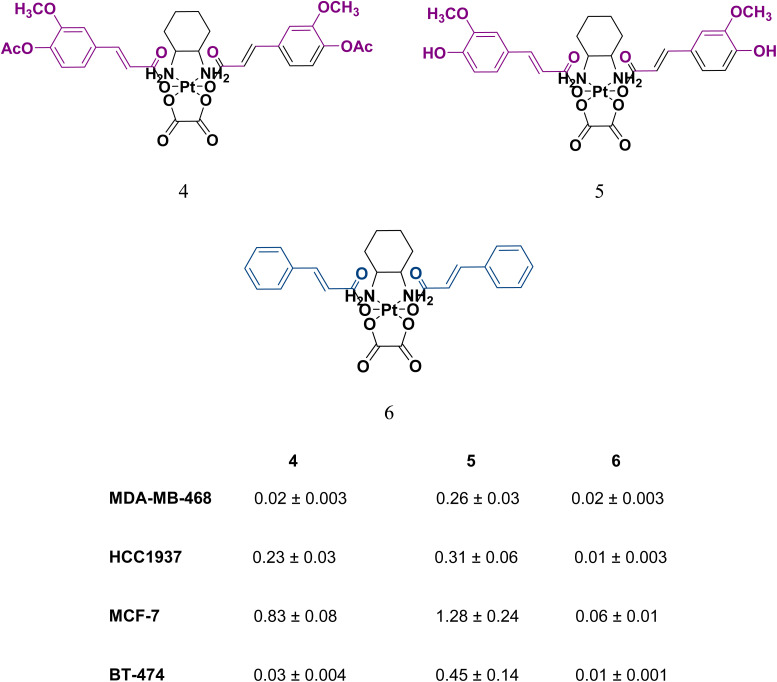
The three oxaliplatin derivatives bearing acetyl-protected (4) and unprotected ferulic acid (5) and cinnamic acid (6), and their corresponding IC_50_ (μm) values on the 4 breast cancer cell lines.

Predarska *et al.*^62^ who once created platinum(iv) complexes from platinum(ii)-based antineoplastic agents (cisplatin) employed the same concept but with oxaliplatin by incorporating in the two axial positions of the platinum(iv) system acetyl-protected and unprotected ferulic acid and cinnamic acid as anionic ligands (Fig. 13) with a DLC% of 8.4%, 8.9%, 11.4%, respectively. The obtained oxaliplatin hybrids were encapsulated with high encapsulation efficiency (ranging from 75 to 97%) into mesoporous SBA-15 material. The produced NPs had a narrow size distribution (200–400 × 600–800 nm). They also showed retarded release of the platinum(iv) hybrids under simulated plasma conditions, suggesting beneficial extended blood circulation with minute drug release. Moreover, only mild release was detected in an acidic pH-simulating stomach. Strong anticancer efficacy was witnessed in four breast cancer cell lines (MCF-7, BT-474, HCC1937, and MDA-MB-468) during the MTT assay, revealing high potential in overcoming oxaliplatin and cisplatin resistance.^65^

A nano-liposomal carrier containing *p*-coumaric acid was prepared by Sabaghi *et al.*^66^ to potentiate its anticancer activity, particularly against melanoma. EE%, size, PDI, and ZP were calculated to be 71.2 ± 0.05%, 109.6 nm, 0.097, and −14.3 mV, respectively. When tested on A375 cells, IC_50_ values of the liposomes were determined to be 62.77 μg mL^−1^ at 24 hours and 55 μg mL^−1^ at 48 hours. Furthermore, flow cytometry analysis revealed that cells treated with the liposome exhibited 5.15% necrosis and 26.96% apoptosis. Regarding the gene expression study, following 48 hours of exposure, the expression of the Bax gene increased, while the expression of both Bcl-2 and TYR genes decreased.^66^

In another attempt to enhance ferulic acid's bioavailability, Sweed *et al.*^46^ loaded it into tocopheryl polyethylene glycol 1000 succinate (TPGS) mixed micelles having an encapsulation efficiency of 99.89%, a PS of 13.86 nm, a PDI less than 0.25, and a ZP of −6.02 mV. *In vitro* assessment by MTT assay and flow cytometry performed on the Caco-2 cell line showed an IC_50_ of 17.1 μg mL^−1^ and induction of cell cycle arrest at the G2/M phase. In addition, a decrease in miRNA 221 levels, an increase in the mRNA expression level of Bax and caspase-3, and gene expression of TP53INP1, all confirmed the mixed micelles' potential for targeting colon cancer *via* the miRNA-221/TP53INP1 axis-mediated autophagy.^46^

El-Adl *et al.*^67^ employed the gamma irradiation-induced polymerization method to synthesize a pH-responsive nanogel system consisting of sodium alginate (NaAlg) and polyacrylic acid (PAAc) loaded with ferulic acid. According to the storage conditions, PS and ZP values were found to be between 59.89 to 89.88 nm and −0.23 and −0.38 mV, respectively. EE% varied from 49.32% to 86.81% according to the pH of the dispersion medium, while the DLC% was around 39%. It was observed that the EE% decreased as the pH increased. MTT assay findings in four cell lines (HepG2, A549, MCF-7, and HCT-116) confirmed the nanogel's potential to impede tumor proliferation. Compared to free ferulic acid, IC_50_ values were reduced by 58.22%, 78.35%, 45.81%, and 47.94% for HepG2, A549, MCF-7, and HCT-116, respectively.^67^

Synthesis and characterization of a biodegradable nano-formulation comprising halogen-coated polymeric pullulan acetate and decorated with ferulic acid was done by Varadhan *et al.*^68^ to address the constraints regarding ferulic acid's stability. The NPs were found to have a PS of 425 ± 5.2 nm, suggesting the ability to target gastrointestinal tract (GIT) tumor cells passively. The EE%, ZP, and PDI were 82.6%, −11 ± 2.4 mV, and 0.6, respectively. Polymeric NPs successfully restrained the growth of GIT's cancer cells (HCT-116, AGS, and SW620), achieving IC_50_ values three times lower than those of free ferulic acid, specifically 55 μg mL^−1^, 60 μg mL^−1^, and 110 μg mL^−1^, respectively.^68^

### 6.4 Nanoparticulate-based caffeic acid

**Fig. 14:**
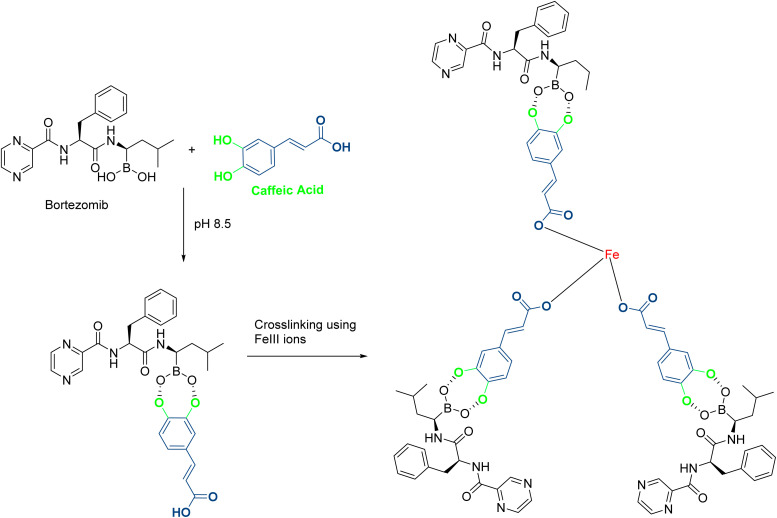
In an alkaline pH, bortezomib complexes with caffeic acid to make a macromolecule that further crosslinks with Fe(iii) ions to stabilize the supramolecular prodrug.

Aguilar *et al.*^69^ developed a supramolecular nanomedicine based on caffeic acid, conjugating it with Bortezomib, a proteasome inhibitor, through a catechol-boronic acid linkage and Fe(iii) ion crosslinking (Fig. 14). Following the dissociation of the catechol–boronic acid linker of the resulting NPs, bortezomib was transported into cancer cells through endocytosis, where it targeted the 26S proteasome, with its effect enhanced by the ROS production from caffeic acid. PS, PDI, and ZP were found to range from 90 to 120 nm and from −20 to −26 mV, respectively, with an inversely proportional relationship over time. Cytotoxicity was validated using CCK-8 and flow cytometry assays on CT26 cells, revealing strong antiproliferative effects and a marked increase in both necrotic and apoptotic cell populations.^69^

**Fig. 15:**
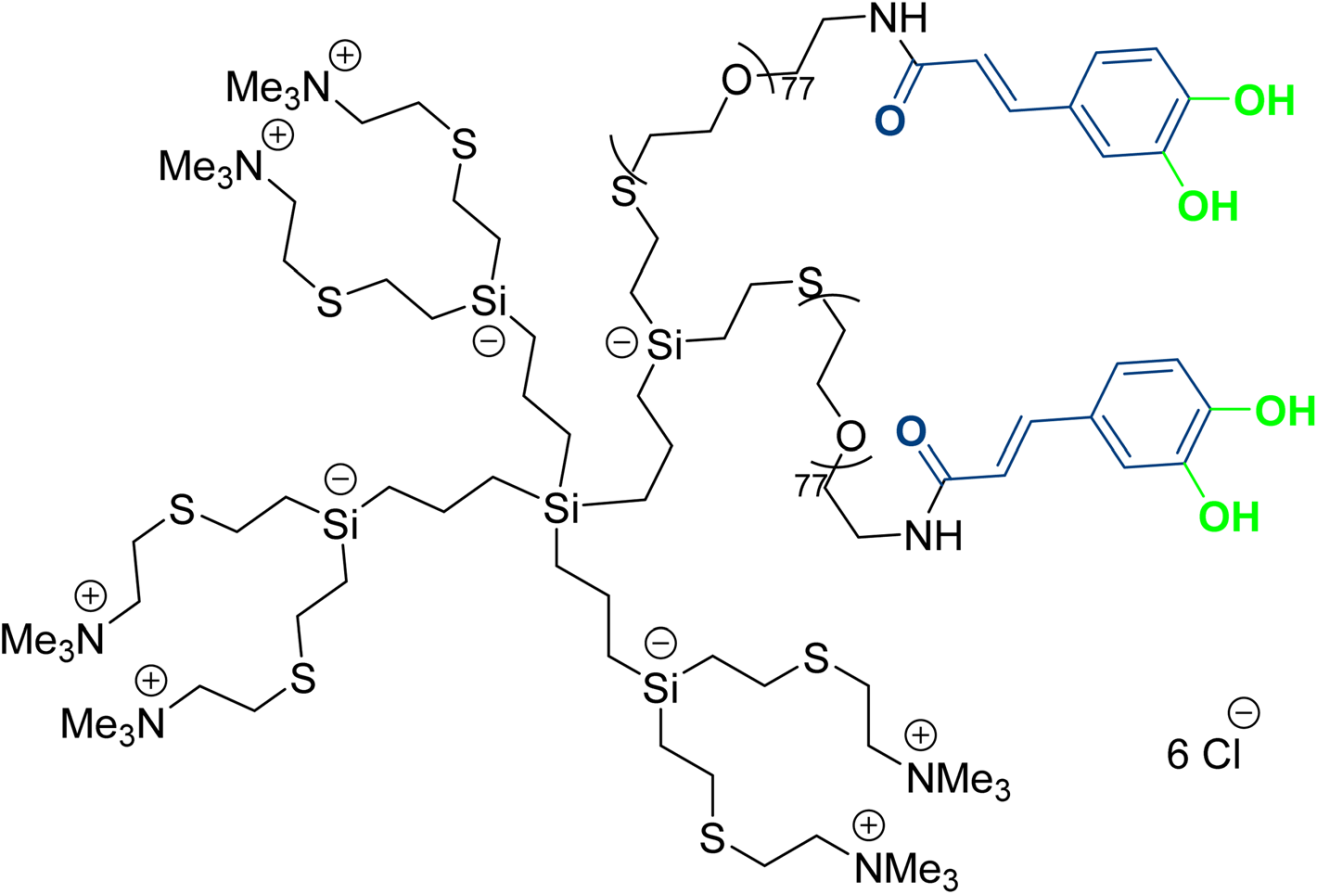
Structure of the designed carbosilane dendrimers with cationic moieties, conjugated to caffeic acid, and bearing PEG groups.

In another work, Grodzicka *et al.*^70^ studied carbosilane dendrimers as a unique drug delivery system and conjugated them with caffeic acid. Two types of dendrimers were developed to enhance the water solubility, one had an ammonium group, and the other was conjugated to PEG (Fig. 15). The NPs showed sizes ranging from 215.6 nm to 476.4 nm with PDI values between 0.44 and 0.6. Ammonium-conjugated dendrimers had a ZP value between 18.3 and 21.6, while dendrimers with anchored PEG had lower ZP values between 2.7 and 2.95 mV. Carbosilane dendrimers exhibited low toxicity toward normal BJ fibroblasts and moderate cytotoxicity against A549 cancer cell lines, reducing cell viability by up to 77% compared to the control at a concentration of 100 μM. Additionally, they exhibited robust antioxidant activity that could help to reduce oxidative hemolysis, lipid peroxidation, and ROS levels.^70^

In 2023, chitosan-grafted carbon nanotubes were loaded with caffeic acid and tested on the MDA-MB-231 cell line for evaluation of their anti-breast cancer activity. MTT assay indicated a dose-dependent growth inhibition of the cell line with an IC_50_ value of 30 μg mL^−1^. This inhibitory effect was mediated through the apoptotic pathway by increased expression of Bax and downregulation of Bcl-2.^71^

Paving the road for a “back to nature” approach, Safwat *et al.*^72^ loaded casein NPs (CS NPs) with caffeic acid and functionalized them with folic acid by simple coacervation followed by lyophilization. CS NPs reached a PS of 157.23 ± 2.64 nm, a PDI of 0.309 ± 0.199, a ZP of −25.53 ± 2.31 mV, and an IC_50_ of 40 ± 2.9 μg mL^−1^ when tested on MCF-7 cancer cells. The EE% was 93.42 ± 3.42%. However, it significantly decreased to 64.30 ± 2.87% after the NPs were exposed to gamma radiation for sterilization purposes. *In vivo* testing on tumor-induced rats showed a downregulation in the level of biochemical markers of Carcino-embryonic antigen, carbohydrate antigen 15–3, and malondialdehyde. Meanwhile, an increased level of superoxide dismutase was spotted. Further investigations of the conjugated NPs are still necessary to advance them into clinical trials.^72^

A recent study utilized caffeic acid to synthesize and stabilize silver NPs (AgNPs), while incorporating it into the NPs. Their analysis showed an average PS of 127.6 nm, a PDI of 0.193, and a ZP of −67.8 mV. Assessment of caffeic acid AgNPs' cytotoxic effect was done by MTT assay on A459 lung cancer cells. Notably, they showed significant toxicity with inhibitory effects observed at concentrations of 141 μg mL^−1^. Cell cycle analysis also confirmed their ability to arrest and kill cancer cells before the synthetic phase without causing any harm to normal cells. Further exploration into their mechanisms of action and possible synergistic interactions with current anticancer treatments may reveal new opportunities for clinical application.^6^

### 6.5 Nanoparticulate-based rosmarinic acid

Fuster *et al.*^73^ designed a rosmarinic acid delivery platform based on silk fibroin NPs and investigated their activity in cervical and breast cancer. The resulting NPs had a PS of 255 nm, a PDI of 0.187, a ZP of −17 mV, a drug loading content of 9.4 weight % %, an EE% of 39%, a DLC% of 9.4 ± 0.5%, and enhanced bioavailability compared to free rosmarinic acid. Cellular death increased in a concentration-dependent pattern following the NPs administration. An IC_50_ of 1.568 and 1.377 mg mL^−1^ on HeLa and MCF-7, respectively, along with decreased tumor growth, were observed in the MTT assay. Cell cycle studies confirmed that the rosmarinic silk fibroin NPs could induce apoptosis, besides inhibiting cellular proliferation.^73^

The solvothermal method was employed by Chircov *et al.*^74^ to obtain two types of magnetic microspheres using PEG as vehicles for the controlled release of rosmarinic acid. One sample had a PS of 218.44 nm, while the other showed a PS of 287.58 nm. ZP values were close to 0 mV for all samples, indicating a tendency for agglomeration. The EE% reached 50.34% after increasing the interaction time between rosmarinic acid and microspheres, while DCL% ranged from 0.5 to 1.83%. The biological assessment using the ROS-Glo H_2_O_2_ assay on BHK kidney cells and the MTT assay on HepG2 cells revealed minimal ROS generation in non-cancerous cells while demonstrating strong antitumor potential against cancer cell lines.^74^

A novel attempt to enhance the bioavailability of rosmarinic acid involved its encapsulation into biodegradable fluorescent tetrasulfide-based porous organosilica NPs. They had a uniform spherical shape, small in size with a PS of 50 nm, a ZP of −13.53 mV, a high DLC% of up to 58.01%, and a stepwise low-dose cargo release. The performed CCK-8 assay showed that administration of NPs containing 140 μg mL^−1^ of rosmarinic acid killed around 39.2% of AGS gastric cancer cells and 36.9% of CT26 cells after 72 hours. Meanwhile, the non-cancerous kidney HEK-293T and NIH-3T3 were spared.^75^

### 6.6 Nanoparticulate-based chlorogenic acid

**Fig. 16:**
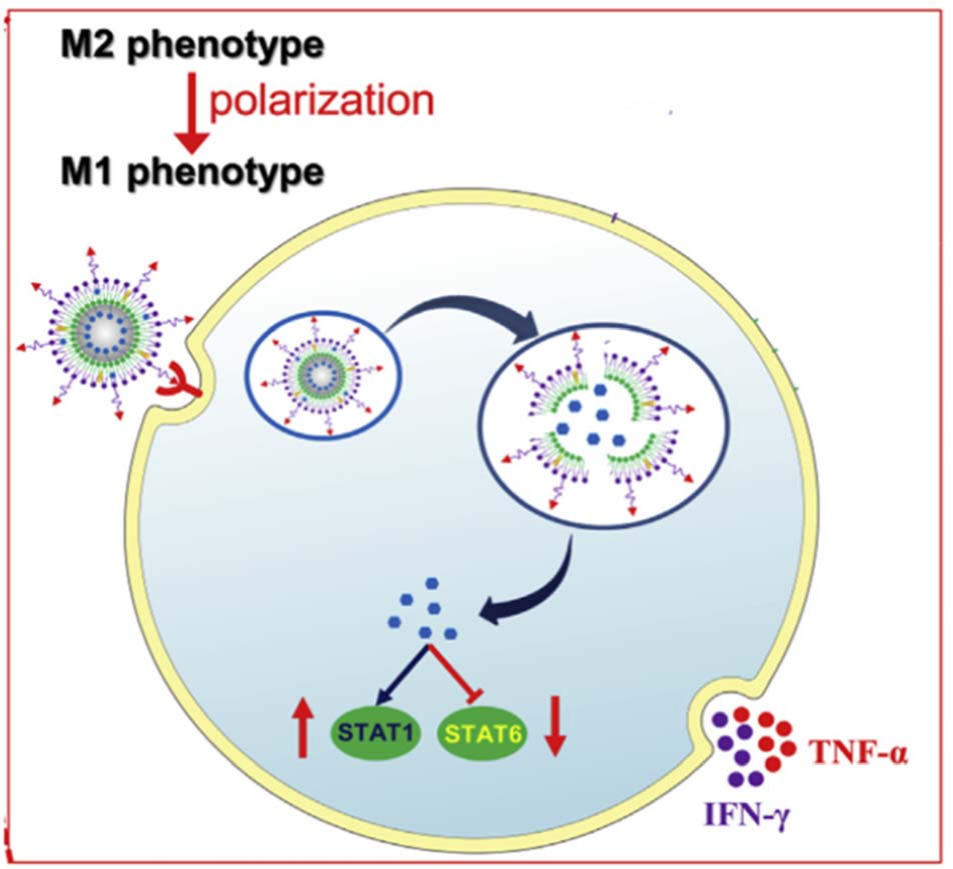
Mannosylated liposomes are efficiently engulfed by TAMs, releasing chlorogenic acid. Once liberated, chlorogenic acid promotes STAT1 activation and inhibition of STAT6 activation, which in turn polarizes M2 into the M1 phenotype. The latter produces immunogenic anticancer cytokines, such as IFN-γ and TNF-α. This figure has been reproduced from ref. 76 permission from Elsevier, copyright (2020).

In general, tumor-associated macrophages (TAMs) show an immunosuppressive M2 phenotype and promote cancer progression. This fact suggests their importance as a target in cancer immunotherapy. Chlorogenic acid has been considered a strong immunomodulator that potentiates the polarization of TAMs from a pro-tumorigenic M2 phenotype to an anti-tumorigenic M1 phenotype that produces immunogenic anticancer cytokines, such as interferon gamma (IFN-γ) and tumor necrosis factor alpha (TNF-α). Therefore, Ye *et al.*^76^ encapsulated chlorogenic acid into mannosylated liposomes effective at targeting TAMs with a PS, PDI, ZP, and EE% of 139.0 ± 0.5 nm, <0.3, −25.3 ± 0.8 mV, and 71.5%, respectively*. In vitro* assays confirmed preferential accumulation of liposomes in tumors and efficient inhibition of G422 glioma tumor growth. Also, the produced liposomes efficiently promoted the conversion of M2 to M1 phenotype (Fig. 16). Thus, the immunotherapeutic efficiency of chlorogenic acid was relatively potentiated by the designed liposomes.^76^

A dual drug nano delivery technique was introduced *via* loading graphene oxide (GO)-PEG nanocomposite with protocatechuic acid and chlorogenic acid. In addition to passive targeting, certain nanocomposites were functionalized with folic acid to enable active targeting. PS, PDI, and ZP of non-coated and folic acid-coated nanocomposites were calculated to be as follows: 34 ± 2.4 nm and 42 ± 3.9; ∼0.28 and ∼0.30, −17.10 ± 1.072 mV and −22.72 ± 2.311 mV, respectively. EE% for protocatechuic acid and chlorogenic acid in non-coated and coated NPs were found to be 75.23%, 72.11%, 79.16%, and 75.15%, respectively, while DLC% was 23.82%, 24.47%, 19.55%, and 23.33%, respectively. Both nanocomposites induced late apoptosis in HepG2 cells with IC_50_ values of 34.73 ± 1.04 μg mL^−1^ (non-coated) and 26.79 ± 1.63 μg mL^−1^ (coated), arrested the cell cycle at the G2/M phase, depolarized mitochondrial membrane potential, and upregulated ROS levels. In addition, no toxic effect was witnessed in the human dermal fibroblast cell line HDFa.^77^

**Fig. 17:**
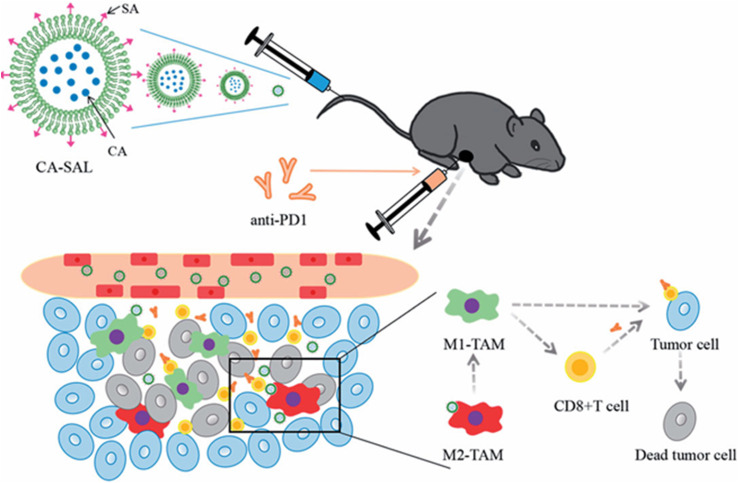
Schematic illustration showing that the tumor mouse model treated with the CA-SAL had a notable reduction in M2-TAMs and CD4^+^Foxp3^+^ T cells, along with an increase in M1-TAMs, CD8^+^ T cells, and T cell activity, resulting in the enhancement of tumor cell death. This figure has been reproduced from ref. 78 permission from Taylor and Francis, copyright (2021). Deed – Attribution 4.0 International – Creative Commons (https://creativecommons.org/licenses/by/4.0/deed.en).

To enhance melanoma immunotherapy, Li *et al.*^78^ combined the anti-programmed cell death 1 (PD-1) antibody with sialic acid (SA) -modified liposomes and loaded with chlorogenic acid (CA-SAL). SA mediated strong cellular uptake *via* SA receptor-mediated TAM targeting. CA-SAL had a PS of 90.36 ± 0.54 nm, a PDI of 0.254 ± 0.006, an EE% of 49.84 ± 2.96%, and a ZP of 13.8 ± 0.1 mV. In the B16F10 melanoma cell line, treatment with the modified liposomes resulted in a notable reduction in M2-TAMs and CD4^+^Foxp3^+^ T cells, along with an increase in M1-TAMs, CD8^+^ T cells, and T cell activity, resulting in the enhancement of tumor inhibition rate (Fig. 17).^78^

**Fig. 18:**
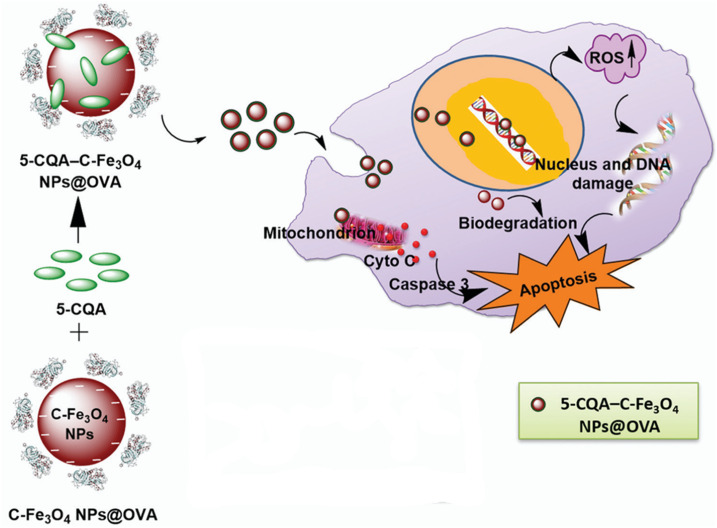
C-Fe_3_O_4_ NPs@OVA were loaded with 5-caffeoylquinic acid (5-CQA). Once inside the cancer cell, they produced ROS that resulted in nuclear damage and increased caspase 3. The latter, along with the DNA damage, led to cell apoptosis. This figure has been reproduced from ref. 79 Permission from RCS, copyright (2022).

The co-precipitation method was used for the preparation of citrate-stabilized Fe_3_O_4_ NPs (C-Fe_3_O_4_) coated with ova-albumin (OVA) and loaded with chlorogenic acid. This design aimed to potentiate chlorogenic acid's anticancer capacity against the MDA-MB-231 cancer cell line. C-Fe_3_O_4_ NPs@OVA had a PS of 52 ± 3 nm, a ZP of −34.1 mV, and an EE% of 91.56 ± 2.1%. They exhibited potent, dose-dependent cytotoxicity with an IC_50_ of 21 ± 1.74 mg mL^−1^, suggesting that the nanocarrier is more likely to invade cells than chlorogenic acid alone. Flow cytometry assay implied tumor growth inhibition was achieved by arresting the cell cycle and producing intracellular ROS (Fig. 18).^79^

Recently, the green fabrication of chlorogenic acid-loaded AgNPs decorated by bovine serum albumin (BSA) took place to verify their plausible antioxidant and antineoplastic effects. The potent *in vitro* antioxidant capability of the NPs was signified by DPPH scavenging and inhibiting lipid peroxidation. PS, PDI, and ZP were 96.51 nm, 0.265, and −17.5 mV. The *in vitro* experimental data showed that AgNPs-BSA had prominent cytotoxicity to Dalton's lymphoma ascites (DLA) cells with an IC_50_ of 2.5 μg mL^−1^, leading to increased ROS levels, and altered levels of oxidized glutathione (GSSG) and reduced glutathione (GSH). Furthermore, *in vivo* experiments demonstrated a significant reduction in tumor cell count, and an increase in serum GSH, CAT, SOD, and glutathione peroxidase activities.^80^

**Fig. 19:**
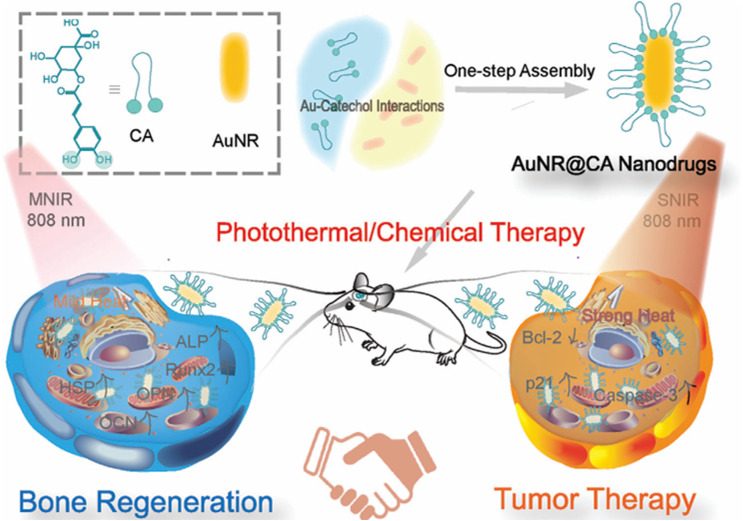
Self-assembly of chlorogenic acid and gold nanorods through gold–catechol interface actions to obtain nanohybrids. Upon exposure to SNIR, nanorods can trigger apoptosis and inhibit tumor growth, while their exposure to MINIR promotes osteogenic differentiation. This figure has been reproduced from ref. 81 permission from Wiley-VCH GmbH, copyright (2023).

Yang *et al.*^81^ initiated a new strategy for assisting osteosarcoma surgical resection by self-assembling chlorogenic acid and gold nanorods (AuNR) through gold–catechol interface actions. The nanohybrids featured adjustable hyperthermia generating capacity, an average size of 23.8 nm, and a loading efficiency of 72.73 wt%. Upon exposure to strong near-infrared (SNIR), the integrated nanorods can severely trigger apoptosis and inhibit tumor growth *in vivo* and *in vitro* on human osteosarcoma cells (Saos-2), reducing cell viability to 0.8% at the highest concentration tested (14.4 μg per mL chlorogenic acid, 3.2 μg per mL AuNR). On the other side, exposure to mild NIR (MINIR) promoted the expression of heat shock proteins and induced significant osteogenic differentiation (Fig. 19).^81^

Alzahrani *et al.*^44^ reported the design, characterization, and exploration of the anticancer activity of albumin–chlorogenic acid (ACNPs) with a PS of 20 nm. Regarding cytotoxicity, several assays took place on MDA-MB-435 breast cancer cells and showed an IC_50_ of 24 μg mL^−1^ after a 24-hour treatment. A significant increase in the comet tail pattern and a substantial upsurge in early apoptotic cells at 24 hours were observed in the comet assay and annexin-V/FITC/PI flow cytometry, respectively. The antioxidant assay revealed a depletion in levels of superoxide dismutase and catalase. In addition, the level of apoptotic proteins, including caspase 3, 8, 9, Bax, cytC, and p53, significantly increased while Bcl-2's level decreased. These apoptotic events could be attributed to a direct mechanism on cellular macromolecules or an indirect one through ROS-mediated oxidative damage. Future research is expected to explore the molecular mechanisms driving breast cancer cell death *in vivo*, utilizing ACNPs as effective alternatives for anticancer therapy.^44^

Li *et al.*^82^ previously synthesized CA-SAL,^78^ combined immunotherapy with chemotherapy by co-loading DOX with chlorogenic acid, hence designated CA-DOX-SAL. CA-DOX-SAL had a PS of 128.3 ± 0.8 nm, a PDI of 0.164 ± 0.003, and a ZP of −4.33 ± 0.50 mV. The EE% for DOX and chlorogenic acid was 94.81 ± 0.42% and 50.24 ± 2.365%, respectively, and the DLC% was 2.7 ± 0.03% and 0.7 ± 0.003%, respectively. Following their administration, CA-DOX-SAL accumulated preferentially in the tumor site, inhibiting its growth. The MTT assay investigating the *in vitro* cytotoxicity of the liposomal formulation in B16F10 and RAW264.7 revealed a reduction in the cell survival in a dose-dependent manner. It exerted a stronger activity than each cargo alone. The activity is driven by two main mechanisms: DOX's direct tumor-killing effect and chlorogenic acid's action on TAMs, reversing the phenotype from tumor-promoting M2-type to anti-tumor M1-type, as previously discussed.^82^

The complex structure of the GIT poses a considerable challenge to the targeted delivery of drugs for colon cancer. Therefore, Zhu *et al.*^13^ developed colon-targeted magnetic-driven orally administered nano vehicles. NPs were made of pectin (PET) and oleic acid-modified iron oxide (Fe_3_O_4_@OA) and encapsulating chlorogenic acid using the combined ultrasound-emulsification technique. The resulting NPs had a uniform PS of 81.04 ± 1.02 nm and resisted degradation by the stomach and small intestine's pH. Once they reached the colon, the pectinase enzyme generated by colonic microbiota facilitated the breakdown of PET, releasing chlorogenic acid. As a result, PET served as a protective barrier, shielding chlorogenic acid from the acidic environment of the GIT. Cytotoxicity CCK8 assay conducted on HCT-116 cancer cells showed a significantly decreased tumor survival rate and an increased chlorogenic acid-killing effect. On the other hand, the hemolysis assay ensured that the NPs were safe and non-toxic in normal cells.^13^

All data regarding the type of nanocarrier, type of cinnamic acid derivative, size, PDI, ZP, EE%, DLC%, target cells, and advantages of nano vehicles encapsulating cinnamic acid and phenylpropanoids are summarized in Table 3.

**Table 3:** Overview of nanoformulations incorporating cinnamic acid and its derivatives, detailing nanocarrier type, physicochemical characteristics (size, PDI, zeta potential, encapsulation efficiency, and drug loading capacity), target cancer cell lines, and reported therapeutic advantages

Type of nanocarrier	Type of cinnamic acid derivative	Size (nm)	PDI	ZP (mV)	EE%	DLC%	Target cells	Advantages	Reference
Dextran NPs	Cinnamaldehyde	166.4	∼0.15	∼−16.4	57.6%	21.6	BEAS-2B cells, HUVEC cells, human cardiomyocytes 4910, and HCT-116 cancer cell line	The NPs showed a pH-responsive release with excellent therapeutic performance and significant *in vivo* safety	52
Cubosomes	Cinnamic acid	N/A	N/A	N/A	N/A	N/A	RAW 264.7 cells and KB cancer cell line	The cubosomes showed higher anticancer activity than free DOX (confirmed by MTT assay) and better cellular uptake (confirmed by flow cytometry assay)	49
Maltodextrin NPs	Cinnamaldehyde	Around 280	Less than 1.2	N/A	N/A	N/A	DU145, SW620, and A549 cancer cell lines	The designed NPs offered a combinational cancer therapy strategy, including oxidative stress inducers and thermal inducers. *In vivo*, they destroyed cancer in mouse xenograft models	53
SLNs	Ferulic acid	174 ± 5	0.166	−25.9	N/A	N/A	HT-29 and NIH 3T3 cells	SLNs had strong cytotoxic activity with an IC_50_ of 10 μg mL^−1^	59
Supramolecular prodrug NPs	Caffeic acid	90–120	N/A	−20 to −26	N/A	N/A	CT26 cells	The NPs showed potent antiproliferative activity, which resulted in a high population of necrotic and apoptotic cells	69
Mannosylated liposomes	Chlorogenic acid	139.0 ± 0.5	<0.3	−25.3 ± 0.8	71.5	N/A	G422 glioma cells	Liposomes potentiated the polarization of TAMs from M2 to M1 phenotype	76
Magnetic (Fe_3_O_4_) NPs functionalized with FITC and folic acid	Cinnamaldehyde	10	N/A	−59.6	N/A	N/A	MCF-7, MDAMB-231, and MCF-10A cells	These NPs prolonged circulation time, enhanced hydrophilicity, and sustained the release of cinnamaldehyde. They had an IC_50_ of 2.84 and 17.44 μg mL^−1^ against MCF-7 and MDAMB-231, respectively	41
Micelles	Cinnamaldehyde	54.3	0.25	N/A	72.8	10.3%	4T1, A549, and HeLa cell lines	The pH-responsive NPs could induce tumor apoptosis with minimal adverse effects on normal tissues. The IC_50_ values were determined to be the CPT-equivalent concentrations 4.6, 3.9, and 3.3 μM, respectively, against the three tested cell lines: 4T1 metastatic breast cancer cells, A549, and HeLa cervical cancer cells	54
Carrier-free self-assembled nano drug particles	Cinnamaldehyde	170	0.121	N/A	N/A	N/A	HepG2, H22, and QSG cell lines	Synergistic anticancer effect (antimetabolic activity by 5FU and generation of ROS by cinnamaldehyde). When tested on the H22 murine hepatoma cell line, 5FU–CA NPs had an IC_50_ of 21.291 μM at 48 hours compared to 66.903 μM for free 5FU and 142.755 μM for free cinnamaldehyde. No significant toxicity was detected on the QSG human hepatocellular cell line	55
pH-sensitive amphiphilic block copolymer NPs	Cinnamaldehyde	N/A	1.33–1.44	N/A	N/A	36.7	A375 and GM cells	The NPs showed a pH-dependent release pattern. They showed enhanced anticancer activity (by cinnamaldehyde and DOX) and promising imaging properties	56
AuNPs	Ferulic acid	34.2 ± 1.3	0.137	−28.5	N/A	N/A	A431 and HaCaT cells	They effectively induced apoptosis, exhibited strong ROS generation, and enhanced caspase-3 activity. The IC_50_ value was calculated to be 168.7 ± 2.3 μg mL^−1^	60
Silk fibroin NPs	Rosmarinic acid	255	0.187	−17	39	9.4 ± 0.5	HeLa and MCF-7 cell lines	Rosmarinic silk fibroin NPs had an IC_50_ of 1.568 and 1.377 mg mL^−1^ on HeLa and MCF-7, respectively. They were capable of inducing apoptosis besides inhibiting cellular proliferation	73
GO-PEG nanocomposite	Protocatechuic acid and chlorogenic acid	Non-coated and coated NPs: 34 ± 2.4 and 42 ± 3.9	Non-coated and coated NPs: ∼0.28 and ∼0.30	Non-coated and coated NPs: ∼0.30, −17.10 ± 1.072 and −22.72 ± 2.311	Protocatechuic acid and chlorogenic acid in non-coated and coated NPs: 75.23, 72.11, 79.16, and 75.15, respectively	Protocatechuic acid and chlorogenic acid in non-coated and coated NPs: 23.82, 24.47, 19.55, and 23.33, respectively	HDFa and HepG2 cells	Both nanocomposites induced late apoptosis in HepG2 cells with IC_50_ values of 34.73 ± 1.04 μg mL^−1^ (non-coated) and 26.79 ± 1.63 μg mL^−1^ (coated), arrested the cell cycle at the G2/M phase, depolarized mitochondrial membrane potential, and upregulated ROS levels	77
SAL	Chlorogenic acid	90.36 ± 0.54	0.254 ± 0.006	13.8 ± 0.1	49.84	N/A	RAW264.7 and B16F10 cell lines	In the B16F10 cell line, treatment with the modified liposomes resulted in a notable reduction in M2-TAMs and CD4^+^Foxp3^+^ T cells, along with an increase in M1-TAMs, CD8^+^ T cells, and T cell activity, resulting in the enhancement of the tumor inhibition rate	78
C-Fe_3_O_4_ NPs@OVA	Chlorogenic acid	52 ± 3	N/A	−34.1	91.56 ± 2.1%	N/A	MDA-MB-231 cancer cell line	C-Fe_3_O_4_ NPs@OVA exhibited potent, dose-dependent cytotoxicity with an IC_50_ of 21 ± 1.74 mg mL^−1^, suggesting that the nanocarrier is more likely to invade cells than chlorogenic acid alone	79
CLC-DOX NPs	Cinnamaldehyde	338	0.164	N/A	N/A	N/A	HepG2 and H22 cell lines	Only 21% of HepG2 and 51.1% of H22 cells survived after treatment with CLC-DOX NPs, demonstrating their enhanced efficacy in killing cancer cells	57
PLGA NPs	Cinnamic acid	186.3	0.047 ± 0.072 to 0.245 ± 0.089	−28.47	76.98	N/A	MDA-MB-231 cell line	CIN-PLGA-NPs exhibited superiority during *in vivo* and *in vitro* evaluation with an IC_50_ of 0.5171 mM *versus* 2.296 mM in cells treated with free cinnamic acid	50
Aptamer-conjugated starch NPs	*p*-Coumaric acid	218.97 ± 3.07	0.299 ± 0.05	29.2 ± 1.35	80.30 ± 0.53	N/A	MDA-MB-231 and NIH3T3 cell lines	The NPs induced cytotoxicity in MDA-MB-231 cells with an IC_50_ value of 22.56 ± 1.97 μg mL^−1^ by regulating ROS levels, causing nuclear damage, altering mitochondrial membrane potential, and inducing apoptosis-related protein expression	45
DTX@PCA NPs	*p*-Coumaric acid	83.4 ± 4.1	∼0.2	−20.4 ± 0.4	33.4 ± 1.3	5.57 ± 0.22	CT26 cell line	DTX@PCA NPs exhibited antiproliferative activity, antimetastatic ability, and enhanced cellular uptake by tumor cells. *In vivo*, they were characterized by preferential tumor accumulation, prolonged systemic circulation, and reduction of DTX side effects	61
MSNs	Cisplatin acetyl-protected caffeate, acetyl-protected ferulate, and ferulate	200–400 × 600–800	N/A	N/A	70.4, 83.8, and 89.2	7.51, 9.23, and 10.33	BT-474, MCF-7, MDA-MB-468, and HCC1937 cell lines	MTT cytotoxicity assay showed excellent anticancer prospects for all three MSNs, with higher cytotoxicity than cisplatin without causing nephrotoxicity	62
PLGA NPs	Ferulic acid	148 ± 6	N/A	6.63 ± 1.3	73 ± 2	N/A	Only tested *in vivo* on a breast cancer mouse model	The NPs repressed Notch synergy with Wnt, estrogen, progesterone, HER2 pathways, P-gp level, and reduced the side-effects of DOX and cyclophosphamide	63
Lipidic NCs	Ferulic acid	36.8	0.2	−12.2	89.8	N/A	Caco-2 and HCT-116 cell lines	Ferulic acid lipid NCs had a potent antioxidant, anti-inflammatory, apoptotic, and cytotoxic potential	64
Carbosilane dendrimers	Caffeic acid	215.6–476.4	0.44–0.6	2.7–2.95	N/A	N/A	BJ and A549 cells	Carbosilane dendrimers exhibited low toxicity toward normal BJ fibroblasts and moderate cytotoxicity against A549 cancer cell lines, reducing cell viability by up to 77% compared to the control at a concentration of 100 μM. Additionally, they exhibited robust antioxidant activity that could help to reduce oxidative hemolysis, lipid peroxidation, and ROS levels	70
Magnetic microspheres	Rosmarinic acid	218.44 and 287.58	N/A	Almost 0	50.34	From 0.5 to 1.83	BHK and HepG2 cells	The microspheres demonstrated strong anti-cancer potential against cancer cell lines while sparing normal tissues	74
AgNP-BSA	Chlorogenic acid	96.51	0.265	−17.5	N/A	N/A	DLA cell line	AgNPs-BSA had prominent cytotoxicity to Dalton's lymphoma ascites (DLA) cells with an IC_50_ of 2.5 μg mL^−1^, leading to increased ROS levels, and altered levels of oxidized glutathione (GSSG) and reduced glutathione (GSH)	80
Porphyrin-based MOF (PCN-224) NPs	Cinnamaldehyde	90	N/A	−22.5	N/A	N/A	Hela and A549 cell lines	The NPs elevated the level of H_2_O_2_, thus improving ROS-mediated cancer cell apoptosis and making PDT/CDT more effective	58
MSNs	Platinum(iv) acetyl-protected and unprotected ferulic acid and cinnamic acid	200–400 × 600–800	N/A	N/A	75–97	8.4, 8.9, and 11.4	MCF-7, BT-474, HCC1937, and MDA-MB-468 cancer cell lines	Overcoming oxaliplatin and cisplatin resistance	65
Chitosan-grafted carbon nanotubes	Caffeic acid	N/A	N/A	N/A	N/A	N/A	MDA-MB-231 cell line	A dose-dependent growth inhibition of the cell line with an IC_50_ value of 30 μg mL^−1^ was detected along with increased expression of bax and downregulation of Bcl-2	71
Nanohybrids	Chlorogenic acid	23.8	N/A	N/A	N/A	N/A	Saos-2 cells	Nanohybrids can severely trigger apoptosis and inhibit tumor growth *in vivo* and *in vitro* on Saos-2 cells, reducing cell viability to 0.8% at the highest concentration tested	81
Albumin NPs	Chlorogenic acid	20	N/A	N/A	N/A	N/A	MDA-MB-435 cells	The NPs showed an IC_50_ of 24 μg mL^−1^ after a 24-hour treatment. A significant increase in the comet tail pattern and a substantial upsurge in early apoptotic cells at 24 hours were observed in the comet assay and annexin-V/FITC/PI flow cytometry, respectively. They also induced a depletion in levels of superoxide dismutase and catalase	44
Zinc oxide NPs	Cinnamic acid	100–200	N/A	N/A	N/A	N/A	KB cells	The obtained NPs showed potent anticancer, antiapoptotic, and antioxidant activity. They resulted in a reduction in cancer cell viability by 97% following their administration	51
Liposomes	*p*-Coumaric acid	109.6	0.097	−14.3	71.2 ± 0.05	N/A	A375 cell line	IC_50_ values of the liposomes were determined to be 62.77 μg mL^−1^ at 24 hours and 55 μg mL^−1^ at 48 hours. Also, cells treated with the liposome exhibited 5.15% necrosis and 26.96% apoptosis	66
TPGS mixed micelles	Ferulic acid	13.86	Less than 0.25	−6.02	99.89	N/A	Caco-2 cell line	Polymeric micelles had the potential for targeting colon cancer *via* the miRNA-221/TP53INP1 axis-mediated autophagy. They had an IC_50_ of 17.1 μg mL^−1^ and induced cell cycle arrest at the G2/M phase	46
Nanogels	Ferulic acid	59.89 to 89.88	N/A	−0.23 to −0.38	49.32 to 86.81	∼39	HepG2, A549, MCF-7, and HCT-116	pH-responsive ferulic acid-loaded nanogels showed a potent cytotoxic effect. Compared to free ferulic acid, IC_50_ values were reduced by 58.22%, 78.35%, 45.81%, and 47.94% for HepG2, A549, MCF-7, and HCT-116, respectively	67
Hyalgan-coated polymeric pullulan acetate NPs	Ferulic acid	425 ± 5.2	0.6	−11 ± 2.4	82.6	N/A	Gastrointestinal cancer cell lines (HCT116, AGS, SW620)	The NPs successfully restrained the growth of GIT's cancer cells (HCT-116, AGS, and SW620), achieving IC_50_ values three times lower than those of free ferulic acid, specifically 55 μg mL^−1^, 60 μg mL^−1^, and 110 μg mL^−1^, respectively	68
Folic acid-functionalized CS NPs	Caffeic acid	157.23 ± 2.64	0.309 ± 0.199	−25.53 ± 2.31	93.42 ± 3.42%	N/A	MCF-7 cell line and tumor-induced rat models	NPs had an IC_50_ of 40 ± 2.9 μg mL^−1^ when tested on MCF-7 cancer cells. A downregulation of the biochemical marker of carcinoembryonic antigen and an increased level of superoxide dismutase were spotted	72
Tetrasulfide-based porous organosilica NPs	Rosmarinic acid	50	N/A	−13.53	N/A	58.01	AGS, CT26, HEK-293T, and NIH-3T3 cells	CCK-8 assay showed that administration of NPs containing 140 μg mL^−1^ of rosmarinic acid killed around 39.2% of AGS gastric cancer cells and 36.9% of CT26 cells after 72 hours	75
SAL	Dox and chlorogenic acid	128.3 ± 0.8	0.164 ± 0.003	−4.33 ± 0.50	DOX: 94.81 ± 0.42 chlorogenic acid: 50.24 ± 2.365	DOX: 2.7 ± 0.03 chlorogenic acid: 0.7 ± 0.003	B16F10 and RAW264.7 cell lines	The MTT assay revealed a reduction in cell survival in a dose-dependent manner. CA-DOX-SAL exerted a stronger activity than each cargo alone	82
PET/Fe_3_O_4_@OA NPs	Chlorogenic acid	81.04 ± 1.02	N/A	N/A	N/A	N/A	HCT-116 cells	PET served as a protective barrier, shielding chlorogenic acid from the acidic environment of the GIT. CCK8 assay showed a significantly decreased tumor survival rate and an increased chlorogenic acid-killing effect	13
AgNPs	Caffeic acid	127.6	0.193	−67.8	N/A	N/A	A549 cells	AgNPs showed significant toxicity, with inhibitory effects observed at concentrations of 141 μg mL^−1^. Cell cycle analysis confirmed NPs' ability to arrest and devastate cancer cells before the synthetic phase	6

## 7 Recent studies regarding encapsulated extracts containing cinnamic acid and the other phenylpropanoids

Other studies reported the anticancer activity of cinnamic acid and its phenylpropanoids in extracts rather than pure compounds. The following section will discuss some examples of nano-formulated natural extracts containing cinnamic acid derivatives.

In a recent study, the aqueous extract of *Deverra tortuosa* (*D. tortuosa*) was used as a reducing and capping agent for the green synthesis of zinc oxide NPs. Upon their synthesis, they had PS values ranging from 9.26 to 31.18 nm. The high-performance liquid chromatography (HPLC) analysis of *D. tortuosa* extract revealed the presence of several polyphenolic compounds responsible for its anticancer activity; many of them were cinnamic acid derivatives, including chlorogenic acid, caffeic acid, coumaric acid, ferulic acid, and cinnamic acid itself. MTT assay of both the extract and zinc oxide NPs showed a concentration-dependent selective cytotoxic activity on the Caco-2 and A549 cell lines, with less cytotoxicity on the normal WI38 lung fibroblast cells. For instance, the IC_50_ values for A549 cells were 193.12 and 83.47 μg mL^−1^ for the extract and the zinc oxide NPs, respectively. For Caco-2 cells, the IC_50_ values were 136.12 μg mL^−1^ for the extract and 50.81 μg mL^−1^ for the zinc oxide NPs. Other biological activities should be assessed and an *in vivo* study should be conducted to corroborate the current study's results.^83^

In 2022, Alshawwa *et al.*^42^ synthesized alpha hematite (α-Fe_2_O_3_) NPs using *Stevia* L. *Rebaudiana* leaf extract (SRLe) as a reducing and stabilizing agent. Analysis of SRL extract's phenolic compounds *via* HPLC showed the presence of several bioactive hydroxycinnamic acid derivatives, including chlorogenic acid, caffeic acid, coumaric acid, and ferulic acid. As a result, the extract had a promising antioxidant radical scavenging activity with an IC_50_ of approximately 13 mg mL^−1^. The resulting SRLe- α-Fe_2_O_3_ NPs had a uniform PS of ∼18.34 nm, a PDI of 0.237, and a ZP of 19.4 mV. Following the cytotoxicity study, they inhibited the proliferation of A549 cell lines in a time-dependent and dose-dependent manner, with an IC_50_ of 51.2 μg mL^−1^, eliminating almost 94% of cancer cells. However, further investigations are needed to determine the precise dosages and reaction conditions necessary for utilizing NPs effectively for these applications.^42^

Mohammed *et al.* employed the medicinal plant *Lycium shawii* and encapsulated it into AgNPs. *Lycium shawii* is characterized by five key metabolites, one of which is the cinnamic acid derivative known as lyciumaside. Lyciumaside is a diacylglyceride widely known for its potent antioxidant properties. The mechanism of action of the five metabolites was predicted using three web servers, which suggested their potential as inhibitors of the carbonic anhydrase IX enzyme. AgNPs had a diameter of 69.43 nm. The cytotoxicity test was performed on three cell lines, HCT-116, MCF-10A, and MDAMB-231. It revealed a potent inhibitory effect against HCT-116 (IC_50_ = 61.74 μg mL^−1^), a weak therapeutic potential against MDAMB-231, and a low toxic effect on the normal cell line.^84^

*Pleurotus ostreatus* (*P. ostreatus*) mushroom has gained significant interest from researchers as a reservoir of secondary metabolites with important biological properties such as anticancer and antioxidant activities. It is well known for its capability of inducing apoptosis and inhibiting angiogenesis. HPLC analysis of *P. ostreatus* extract (PE) revealed the presence of several phenolic compounds, including cinnamic acid and many of its derivatives like chlorogenic acid, caffeic acid, coumaric acid, and ferulic acid. Hence, Al-Rajhi *et al.*^9^ loaded PE into chitosan NPs (PELchNPs) and compared their anticancer activity against MCF-7 cancer cell lines with the free extract. PELChNPs demonstrated significantly higher effectiveness against the MCF-7 cell line than PE, especially at lower concentrations. The proliferation of the MCF-7 cell line was suppressed at concentrations of 1 μg mL^−1^ for PELChNPs and 3.9 μg mL^−1^ for PE. The IC_50_ value further highlighted the potency of PELChNPs, which was notably more effective than Vinblastine sulfate, having an IC_50_ of around 6 μg mL^−1^.^9^

Among the natural products that substantially positively impact health and medicine, bee pollen is characterized by a high concentration of proteins, polyphenols, minerals, and vitamins. Chlorogenic, caffeic, coumaric, and cinnamic acids were all isolated from bee pollen. Thereby, Hanafy *et al.*^85^ isolated the polyphenolic compounds and incorporated them together with bevacizumab into a hybrid peptide–protein hydrogel NPs. The latter were made through combining bovine serum albumin with protamine-free sulfate and targeted with folic acid. The NPs exhibited a mean diameter of 138.2 nm, a polydispersity index (PDI) of 0.3, a zeta potential of +17.6 ± 2 mV, and an encapsulation efficiency (EE%) of 98 ± 0.23%. Their pro-apoptotic effects were evaluated using A549 and MCF-7 cancer cell lines. The MTT assay revealed half-maximal inhibitory concentrations (IC_50_) of 214.3 μg mL^−1^ for A549 cells and 125.6 μg mL^−1^ for MCF-7 cells. Also, significant upregulation of Bax and caspase 3 genes and downregulation of Bcl-2, HRAS, and MAPK were recorded. As a result, simultaneous administration of these NPs with chemotherapies would potentiate the chemotherapeutic effect and minimize the required dose.^85^

In 2024, Abdelaziz *et al.*^86^ also developed new chitosan NPs (CsNPs) encapsulating star anise extract (SAE) with optimized abilities to fight lung cancer. SAE contains cinnamic acid and many of its hydroxy derivatives with different concentrations, accounting for its medicinal quality. The bioactive derivatives detected by HPLC analysis of SAE included chlorogenic acid, caffeic acid, coumaric acid, ferulic acid, and rosmarinic acid. SAE-encapsulated CsNPs had an average PS of 318.5 ± 73.94 nm and a homogenous distribution with a PDI value of 0.547. MTT assay was used to assess the cytotoxic effect of the prepared NPs against the NCl-H460 lung cancer cell line. The latter's viability was reduced in a dose-dependent way, reaching 38.2% for SAE-loaded CsNPs at 100 μg mL^−1^. Meanwhile, *in vivo* studies demonstrated a reduction in tumor-associated biomarkers and inflammatory mediators, including malondialdehyde (MDA), p53, TNF-α, and fibronectin.^86^

The bee product propolis is widely known for its anticancer, antioxidant, and antibacterial activity. It has been used for ages as a dietary supplement due to its rich content of bioactive ingredients. Caffeic acid, ferulic acid, cinnamic acid, coumaric acid, and caffeic acid phenethyl ester are among the main phenolic components of propolis. Regarding its anticancer activity, several articles reported the incorporation of propolis extract into different types of NPs to enhance its potential and overcome its bioavailability issues, and were summarized in multiple reviews. For instance, chitosan-coated nano propolis with a PS of less than 200 nm could ameliorate the side effects of cisplatin, including liver and kidney damage.^87^ In addition, propolis-loaded niosomes showed anticancer activity against lung cancer. PS, PDI, ZP, and EE% were calculated to be 151 ± 2.84 nm, 0.232, −30.9 ± 0.33 mV, and 70%. They decreased cell viability to below 50% after 24 h of treatment and the scattering in the 3D spheroids of A549 cells.^88^ In 2023, propolis NPs had a prominent apoptotic effect on breast cancer. Their IC_50s_ were recorded to be 13.67 ± 0.89, 17.89 ± 0.6, and 29.9 ± 0.56 μg mL^−1^ on MCF-7, MDA-MB-231, and MCF-10A cells, respectively. They showed a PS of 59.28 nm, a PDI of 0.507, and a ZP of −4.21 mV.^89^ Furthermore, propolis-loaded NLC halted breast cancer progression. The latter took place through different mechanisms, all related to an elevated level of miRNA-223 expression. The lipid carrier had a PS, a PDI, a ZP, and an EE% of 255.8 ± 0.67 nm, 0.263 ± 0.009, −28.3 ± 4.71 mV, and 87.70 ± 2.82% respectively.^90^ In addition, propolis zinc oxide NPs exhibited potent antioxidant and anticancer properties. They had a PS of 9.70 nm and a ZP of −27 mV. An IC_50_ of 18 and 23 μg mL^−1^ was witnessed for MCF-7 and HelA cell lines, respectively.^91^ Likewise, *propolis* extract nanocapsules containing supercritical fluid carbon dioxide extracts revealed their significant potential as natural antioxidants and anticancer agents with a PS ranging from 1.49 to 36.14 nm and an EE% of 95.89%. They exhibited a high cytotoxic effect with IC_50_ values of 99.2, 124.6, and 90.8 μg mL^−1^ against PC-3 (prostate cancer), MCF-7, and HePG-2 cell lines, respectively.^92^

All data regarding the type of nanocarrier, type of cinnamic acid derivative, size, PDI, ZP, EE%, DLC%, target cells, and advantages of nano vehicles encapsulating extracts containing cinnamic acid and phenylpropanoids are summarized in Table 4.

**Table 4:** Summary of *in vitro* and *in vivo* anticancer evaluations of nanoformulations encapsulating cinnamic acid and its derivatives, including key physicochemical parameters, tested cell lines, IC_50_ values, and therapeutic outcomes

Type of nanocarrier	Type of cinnamic acid derivative	Size (nm)	PDI	ZP (mV)	EE%	DLC%	Target cells	Advantages	Reference
Zinc oxide NPs	*D. tortuosa* extract, including chlorogenic acid, coffeic acid, coumaric acid, ferulic acid, and cinnamic acid	From 9.26 to 31.18	N/A	N/A	N/A	N/A	Caco-2, A549, and WI38 cell lines	The IC_50_ values for A549 cells were 193.12 and 83.47 μg mL^−1^ for the extract and the zinc oxide NPs, respectively. For Caco-2 cells, the IC_50_ values were 136.12 μg mL^−1^ for the extract and 50.81 μg mL^−1^ for the zinc oxide NPs	83
α-Fe_2_O_3_ NPs	SRL extract containing chlorogenic acid, caffeic acid, coumaric acid, and ferulic acid	∼18.34	0.237	19.4	N/A	N/A	A549 cell lines	SRLe- α-Fe_2_O_3_ NPs inhibited the proliferation of A549 cell lines in a time-dependent and dose-dependent manner, with an IC_50_ of 51.2 μg mL^−1^, eliminating almost 94% of cancer cells	42
AgNPs	Lyciumaside	69.43	0.385	N/A	N/A	N/A	HCT-116, MCF-10A, and MDAMB-231 cell lines	Potent cytotoxicity against the HCT-116 (IC_50_ = 61.74 μg mL^−1^) cell line was witnessed	84
Chitosan NPs	Chlorogenic acid, caffeic acid, coumaric acid, ferulic acid, and cinnamic acid	N/A	N/A	N/A	N/A	N/A	MCF-7 cell line	PELChNPs demonstrated significantly higher effectiveness against the MCF-7 cell line than PE, especially at lower concentrations. The proliferation of the MCF-7 cell line was suppressed at concentrations of 1 μg mL^−1^ for PELChNPs and 3.9 μg mL^−1^ for PE. The IC_50_ value further highlighted the potency of PELChNPs, which was notably more effective than Vinblastine sulfate, having an IC_50_ of around 6 μg mL^−1^	9
Hydrogel NPs	Chlorogenic acid, caffeic acid, coumaric acid, and cinnamic acid	138.2	0.3	17.62	98 ± 0.23	N/A	A549 and MCF-7 cell lines	Hydrogel NPs had an IC_50_ of 214.3 μg mL^−1^ and 125.6 μg mL^−1^ on A549 and MCF-7 cells, respectively. They significantly upregulated Bax and caspase 3 genes, and downregulated Bcl-2, HRAS, and MAPK	85
Chitosan NPs	SAE extract includes chlorogenic acid, caffeic acid, coumaric acid, ferulic acid, rosmarinic acid, and cinnamic acid	318.5 ± 73.94	0.547	N/A	N/A	N/A	NCl-H460 cell line	The viability of NCI-H460 cells decreased in a dose-dependent manner, dropping to 38.2% at a NP concentration of 100 μg mL^−1^	86
								Also, a significant reduction in tumor biomarkers and inflammation was observed during *in vivo* studies	
Chitosan	Propolis	Less than 200 nm	N/A	N/A	N/A	N/A	Only tested *in vivo* on Sprague-Dawley rat models	The NPs ameliorated cisplatin's side effects	87
Niosomes		151 ± 2.84	0.232	−30.9 ± 0.33	70	N/A	A549 and BEAS-2B cells	They decreased cell viability to below 50% after 24 h of treatment, and the scattering in the 3D spheroids of A549 cells	88
Propolis NPs		59.28	0.507	−4.21	N/A	N/A	MCF-7, MDA-MB-231, and MCF-10A cell lines	The IC_50_ values recorded on MCF-7, MDA-MB-231, and MCF-10A cells were 13.67 ± 0.89, 17.89 ± 0.6, and 29.9 ± 0.56 μg mL^−1^, respectively	89
NLC		255.8 ± 0.67	0.263 ± 0.009	−28.3 ± 4.71	87.70 ± 2.82%	N/A	Tested only *in vivo* in Ehrlich ascites carcinoma-bearing mice	They halted breast cancer progression through different mechanisms, all related to an elevated level of miRNA-223 expression	90
Zinc oxide NPs		9.70	N/A	−27	N/A	N/A	MCF-7 and HeLa cell lines	An IC_50_ of 18 and 23 μg mL^−1^ was witnessed for MCF-7 and HeLa cell lines, respectively	91
Propolis nanocapsule		1.49–36.14	N/A	N/A	95.89	N/A	PC-3, MCF-7, and HePG-2 cell lines	They had a high cytotoxic effect with IC_50_ values of 99.2, 124.6, and 90.8 μg mL^−1^ against PC-3, MCF-7, and HePG-2 cell lines, respectively	92

## 8 Conclusion and future directions

Cinnamic acid and its derivatives represent a promising class of natural anticancer agents with diverse pharmacological properties. However, their clinical translation is hindered by critical limitations such as poor aqueous solubility, limited bioavailability, rapid systemic clearance, and non-specific distribution. This review has provided a comprehensive overview of recent advances in the nanoformulation of cinnamic acid-based compounds, demonstrating how nanoparticle-based delivery systems can address these shortcomings. By improving drug solubility, prolonging systemic circulation, enhancing tumor accumulation *via* the enhanced permeability and retention (EPR) effect, and reducing off-target adverse effects, nanotechnology has significantly elevated the therapeutic potential of these compounds. Despite this progress, several challenges remain before these nanoformulations transition from bench to clinic. Future research should prioritize the scalability and reproducibility of synthesis methods, alongside in-depth pharmacokinetic and biodistribution studies in relevant animal models. A critical step forward will be the systematic evaluation of biosafety and long-term toxicity, as well as the immunological interactions of these nanosystems within the tumor microenvironment. Moreover, there is an urgent need to progress beyond *in vitro* characterization and move toward robust *in vivo* models and early-phase clinical trials. Moreover, special focus should be given to inventing multifunctional nanocarriers capable of active targeting, stimuli-responsive release, and integration with other therapeutic modalities such as immunotherapy or photodynamic therapy. Ultimately, advancing cinnamic acid-loaded nanocarriers toward clinical application will require a multidisciplinary strategy that bridges chemistry, oncology, immunology, and nanomedicine.

## Conflicts of interest

There are no conflicts to declare.

## Data Availability

This manuscript does not involve any experimental work.
